# One Health Determinants of *Escherichia coli* Antimicrobial Resistance in Humans in the Community: An Umbrella Review

**DOI:** 10.3390/ijms242417204

**Published:** 2023-12-06

**Authors:** Chloé C. H. Smit, Maarten Lambert, Kris Rogers, Steven P. Djordjevic, Antoine M. Van Oijen, Caitlin Keighley, Katja Taxis, Hamish Robertson, Lisa G. Pont

**Affiliations:** 1 Graduate School of Health, University of Technology Sydney, Sydney, NSW 2008, Australia; chloe.smit@student.uts.edu.au (C.C.H.S.); kris.rogers@uts.edu.au (K.R.); 2 Department of PharmacoTherapy, -Epidemiology and -Economics, Faculty of Science and Engineering, University of Groningen, 9713 AV Groningen, The Netherlands; m.lambert@rug.nl (M.L.); k.taxis@rug.nl (K.T.); 3 The Australian Institute for Microbiology & Infection, University of Technology Sydney, Sydney, NSW 2007, Australia; steven.djordjevic@uts.edu.au; 4 Faculty of Medicine and Health, University of Sydney, Sydney, NSW 2050, Australia; antoine.vanoijen@sydney.edu.au; 5 Southern.IML Pathology, Sonic Healthcare, 3 Bridge St, Wollongong, NSW 2500, Australia; 6 Graduate School of Medicine, University of Wollongong, Wollongong, NSW 2522, Australia; 7 School of Public Health & Social Work, Queensland University of Technology, Brisbane, QLD 4059, Australia; h5.robertson@qut.edu.au

**Keywords:** antimicrobial resistance, antibiotics, One Health, risk factor, community, human, *Escherichia coli*

## Abstract

To date, the scientific literature on health variables for *Escherichia coli* antimicrobial resistance (AMR) has been investigated throughout several systematic reviews, often with a focus on only one aspect of the One Health variables: human, animal, or environment. The aim of this umbrella review is to conduct a systematic synthesis of existing evidence on *Escherichia coli* AMR in humans in the community from a One Health perspective. PubMed, EMBASE, and CINAHL were searched on “antibiotic resistance” and “systematic review” from inception until 25 March 2022 (PROSPERO: CRD42022316431). The methodological quality was assessed, and the importance of identified variables was tabulated across all included reviews. Twenty-three reviews were included in this study, covering 860 primary studies. All reviews were of (critically) low quality. Most reviews focused on humans (20), 3 on animals, and 1 on both human and environmental variables. Antibiotic use, urinary tract infections, diabetes, and international travel were identified as the most important human variables. Poultry farms and swimming in freshwater were identified as potential sources for AMR transmission from the animal and environmental perspectives. This umbrella review highlights a gap in high-quality literature investigating the time between variable exposure, AMR testing, and animal and environmental AMR variables.

## 1. Introduction

Antimicrobial resistance (AMR) is a global problem leading to untreatable infections that occurs by natural selection but is driven by antibiotic exposure in healthcare (humans), agriculture (animals, plants, or food-processing technology), and the environment (sea, soil, drinking water, and wastewater) [1,2,3,4]. The use of antibiotics in humans and animals is perceived as the major contributor to the development of AMR [5]. With AMR increasing and new antibiotic development stagnating, problems due to untreatable infections can be expected to increase health-related burdens, including more extended hospital stays, increased healthcare costs, and death [6]. Investigating the interaction between humans, animals, and the environment, as well as between the different sectors involved (e.g., pharmaceutical industry, food industry, water waste companies), using a One Health approach, is of great importance in mitigating resistance [7].

*Escherichia coli* (*E. coli*) is a common commensal of the intestinal microbiota in both animals and humans [8,9] that has received significant attention in the literature [10,11] due to increasing AMR [12,13] and death associated with resistance [14,15]. *E. coli* infections are caused by extraintestinal and uropathogenic subtypes [16], with uropathogenic *E. coli* responsible for up to 80% of urinary tract infections [17], the most common infectious disease in the community [18]. Virulence potential varies according to molecular types of bacterial isolates [19]. AMR of *E. coli* is due to both intrinsic (the outer membrane and expression of efflux pumps) and extrinsic mechanisms (the acquisition of mobile genetic elements or through horizontal gene transfer that assists in capturing, accumulating, and disseminating resistance genes [20]). New antimicrobial resistance genes continuously emerge, leading to multidrug resistance [21,22]. *E. coli* can mobilize resistant genes more easily than other bacteria populations and act as a reservoir for AMR genes and mobile genetic elements, and is mainly driven by external factors [12,20]. It is, therefore, essential to understand the community variables leading to AMR of *E. coli*.

To establish evidence around AMR development, there is a need for a clear understanding of association or predictive and temporal relationships between variables. In this research, we have used the term “variable” to describe causal variables, risk factors, and confounders [23,24]. Furthermore, the definition of AMR is widespread, resulting in different interpretations and outcomes from clinicians and public health perspectives [25]. AMR is investigated in symptomatic populations (e.g., the emergence of infection, colonization) for pathogenic bacteria, in asymptomatic people (e.g., carriage, acquisition and transmission) for commensal bacteria and by molecular investigations (e.g., resistant genes) in humans, animals, and the environment [26,27]. Therefore, when reviewing the literature on variables of AMR, a broad perspective should be considered.

Variables known to be associated with AMR have been identified in multiple systematic reviews, but most are focused on the hospital setting, only one aspect of the one health perspective or based in one country/region [28,29,30,31,32]. The purpose of the present study is to provide a comprehensive and systematic overview of the literature to assess the importance and evidence related to variables for resistance and the temporal relationship between variables and resistance development for the community through an umbrella review. The umbrella review methodology allows a bird’s-eye view of the association between human, animal, environmental, and temporal relationships between variables and resistance [33]. An umbrella review aims not to repeat searches, assess study eligibility, risk of bias assessment, or perform a meta-analysis from the included systematic reviews, but to provide an overall picture of the findings for a particular phenomenon [33].

## 2. Results

**Figure 1:**
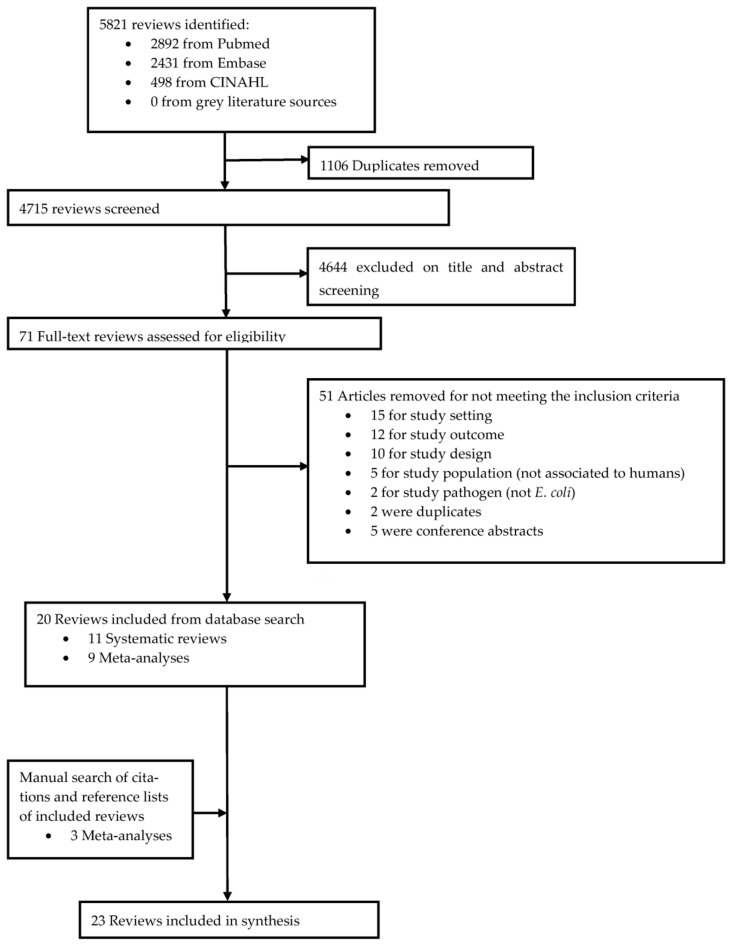
Flowchart of systematic reviews included in the umbrella review.

We identified 5823 reviews, from which 1106 duplicates were removed. Seventy-one reviews were identified for full-text assessment, and twenty studies were eligible for inclusion (Supplementary Table S1). Screening reference lists and citations of those reviews resulted in three additional studies. No other studies were found through CoCites or the websites of key organizations, giving a total of 23 reviews (Figure 1).

### 2.1. Review Characteristics

The 23 reviews included 860 primary studies (Table 1). Nineteen reviews focused on human variables of AMR, three focussed on animal-related variables of AMR, and one looked at variables of AMR in humans and the environment. Geographically, most reviews investigated variables in Europe or North America (18/23), whereas only eight reviews investigated variables in Africa or Oceania.

### 2.2. Quality Assessment

The quality of all included studies was rated as critically low, with Willems et al. [55] as the sole exception, with a low-quality rating (Supplementary Table S2). The main issues affecting the methodological quality of included reviews were not explaining the choice of study design, not reporting on the funding of included studies, not assessing the impact of risk of bias of individual studies, and/or not accounting for the individual risk of bias.

### 2.3. Human Variables

#### 2.3.1. Antibiotic Use

Of the human-related variables, antibiotic use was most frequently reported as a variable for AMR (Table 2). Most reviews investigating the impact of antibiotic use on AMR *E. coli* reported a positive association ranging from general antibiotic use increasing the odds by 1.5 and use of fluoroquinolones increasing the odds by 19 times (Table 2). Longer duration of use was associated with increased odds of AMR *E. coli*, as was the use of multiple courses and mass administration across populations such as HIV-infected adults and young children. The use of β-lactam antibiotics was identified as the most important variable in this category, followed by (fluoro)quinolone- and cephalosporin antibiotics [48]. There were no [15,46] statistical results reported around sulphonamides, trimethoprim [35,42,52], and tetracycline [37,53] use.

#### 2.3.2. Comorbidities, Medication Use, and Hospitalization

Urogenital comorbidities increased the odds of AMR *E. coli*, as did some non-urogenital conditions (Table 2), with the most important variables being previous/recurrent urinary tract infection (UTI) [48] and diabetes [48]. There were mixed results for variables indicating increased vulnerability, with a positive association for previous hospitalization [46] and corticosteroid use [48], mixed results for acid suppressants [15,55], and no association for increased odds of AMR *E. coli* in those with chronic disease [15] or renal and urological disorders [48].

#### 2.3.3. Diet, Sex, Age, and Living

Vegetarian diet, older age (>55 years) [48], and children attending day-care [41] increased the odds of AMR *E. coli* varying from 1.5 to 2.0 (Table 2). Raw milk [15] and lower socioeconomic status [34] were found to be the most important variables in this category. A weekly fish meal and living in Northern Europe compared to Southern Europe were found to reduce the risk of infection of AMR *E. coli* [48] (Table 3).

#### 2.3.4. Travel

The last human-related variable was travel, with destination, health while traveling, traveler demographics, protective measures, and household transmission as subcategories (Table 3). International travel [46,48] increased the odds of AMR *E. coli*, with Asia [15,45,54] and India [15,46] as travel destinations having the highest risks and were found to be the most important variables in this category. Reviews reporting on bowel-related diseases while traveling reported a positive association with odds for AMR *E. coli* ranging from 1.6 [15] to 31 [45]. Antibiotic use while traveling showed a positive association in all reviews, increasing odds from 2.4 [43] to 5 [45]. There were no conclusive results around food consumption while traveling on the odds of AMR *E. coli*, with a vegetarian diet increasing the odds by 1.4 [43], raw vegetable consumption showing mixed results and odds after street food consumption varying from approximately 1.4 to 2.1 [15]. Protective measures while traveling were proven ineffective [43,54]. International travel, followed by travel to Asia, travel to India, antibiotic use while traveling, vegetarian diet, and street food consumption were identified as important variables.

### 2.4. Animal and Environmental Variables

Of the animal-related variables, pets and farming were investigated in reviews for increasing the odds of AMR *E. coli* amongst community-dwelling populations (Table 4). All reviews reporting on pet owners reported no increased odds of AMR *E. coli*. No statistical results were reported on farming. Amongst the types of farms, poultry in the Netherlands has been identified as a probable source of genetic AMR *E. coli* transmission in two reviews identified through whole-genome sequencing [47,49]. Looking at the environmental-related variables, swimming in freshwater doubled the risk of AMR *E. coli* infection in one systematic review [48] (Table 4). No variables were identified as important in both categories.

### 2.5. Temporal Relationship Variable and AMR E. coli

Eleven reviews investigated the temporal relation of variables and outcomes of AMR *E. coli* with antibiotic use and travel as subcategories (Table 5). Reviews showed that resistance after antibiotic use can persist for up to 12 months [15,39,42]. All cut-off points before one year were consistently associated with increasing the odds of AMR *E. coli* varying from 1.4 to 13.2. The risk of AMR *E. coli* after traveling abroad is highest in the first six weeks but decreases over time [43]. Six months [39,51] after antibiotic use was identified as the most important variable for AMR *E. coli*, followed by one and three months [38,39,51].

## 3. Discussion

**Figure 2:**
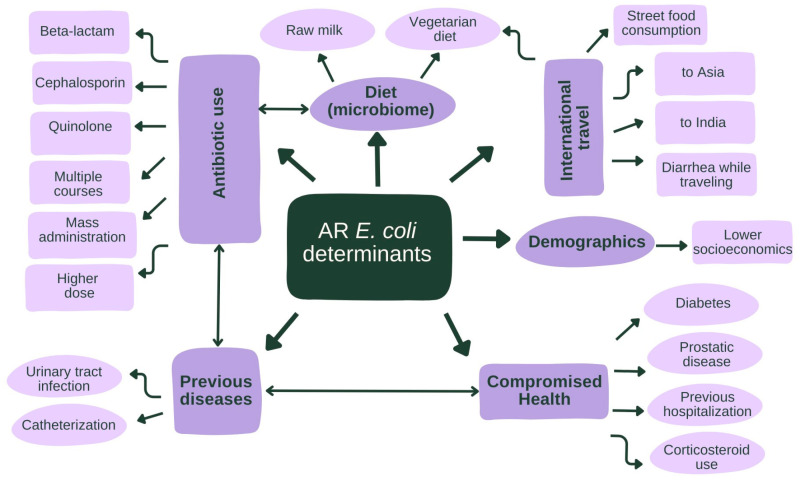
Graphical visualization of the most important human variables and subcategories identified in this umbrella review, with ←→ indicating the relationship between variable groups. Antibiotic use is both a variable for AMR *E. coli* and a potential cause and/or the result of other variables, including gut microbiome interference, previous disease, and compromised health.

In this review, we identified the following human variables for AMR *E. coli*: antibiotic use, comorbidities (recurrent or previous UTI, catheterization, diabetes, prostatic disease), corticosteroid use, previous hospitalization, diet (raw milk and vegetarian), lower socioeconomic status, and international travel (Figure 2). Poultry farms and swimming in freshwater were identified as potential animal and environmental variables for AMR of *E. coli.* We identified a temporal relationship for AMR *E. coli* 6 weeks after travel and up to 12 months after antibiotic use. Living in Northern Europe versus Southern Europe and eating a weekly fish meal were found to be protective against AMR *E. coli*.

There is a large body of literature on variables of the human aspect of the One Health perspective, but environmental and animal aspects have not been studied equally. Like our study, Campos-Madueno et al. found no and weak evidence for transmission between pets and farm animals [56]. Wild animals, particularly birds, can carry AMR *E. coli* strains in the gut by obtaining food in polluted environments [57]. While research into animals and environmental variables is limited, a scoping review on variables from a One Health perspective in Latin America found antibiotic use in animals and the role of food from animal origin to be the most frequent animal contributors to AMR, with wastewater, soil, farm/bird coops, vectors (flies), and pond sediments identified as environmental contributors to AMR spread [32]. Climate change, mainly the increase in temperature, has also been identified as a potential environmental variable. However, this might be a proxy for higher antibiotic use [58]. Swimming in freshwater in Norway was identified as the only environmental variable, which may show the possible link between antibiotic pollution of the environment and AMR gut colonization. A UK study investigating surfer gut colonization also found an increased risk for gut colonization by AMR *E. coli* in surfers compared to non-surfers [59]. Controversially, eating fish was protective in one study executed in Norway. Different levels of antibiotics in water compared to fish farming might explain such differences [60]. A recent systematic review stated incomplete understanding of AMR acquisition and spread between humans, animals, and the environment [61]. Tracing the direction of AMR transfer is important but particularly difficult in cases where antibiotics have been used in animals, plants (environment) and humans [5]. Genetic testing of molecular types can assist in tracing the direction and the virulence of AMR [19].

Antibiotics, drinking raw milk, and a vegetarian diet are all known to affect the microbiome in the gut. Interference of the gut microbiome might increase the risk of a commensal *E. coli* becoming pathogenic [62]. As diet generally affects the microbial flora in the gut, it could be that diet may be indicative of other variables not yet measured. Other variables related to increased infection (risk), such as (traveler’s) diarrhea, previous UTI, catheterization, diabetes, corticosteroid use, and healthcare exposure, might be a proxy for antibiotic use/prophylaxis (Figure 2).

The impact of the geographical location not only identified in our review but also in those investigating molecular epidemiology of resistant *E. coli* genes [21,22] is based on different factors, including sanitation and hygiene practices, antibiotic use regulations, and level of antibiotic pollution [63]. Living in Northern Europe, a region with strict antibiotic use policies, was a protective factor. Furthermore, the overall prevalence of carriage of AMR *E. coli* in healthy populations varies geographically, with intestinal colonization of ESBL *E. coli* highest in Southeast Asia (27%) and lowest for Europe (6%), indicating higher risk after traveling to Asia (Figure 2) [64]. In our umbrella review, limited evidence is collected from Africa or Oceania.

The reduction of not only antibiotic use but also antibiotic pollution of the environment may assist in the reduction and spread of *E. coli* AMR in humans in the community. Furthermore, policies focused on (genetic) screening of humans and animals before travel and environmental sources such as water waste and wild animals’ feces are needed to identify the magnitude of the problem and assist in where to intervene for future *E. coli* AMR spread.

A key strength of this review was the umbrella review methodology, which enabled a bird’s-eye overview of all variables to date on AMR *E. coli* and identified important gaps in the literature. However, this research also had limitations. We could not fully explore the temporal relationship between variables and resistance as the time between variable exposure and measurement of resistance has not been reported in all reviews. Moreover, even though resistance can occur quickly in a lab, there can be a delay in the emergence of detectable resistance clinically in the community [5]. Secondly, some of the variables had wide confidence intervals, probably due to the large heterogeneity of groups within the studies included in the systematic reviews. Lastly, the reviews included in this umbrella review were of critically low quality and were diverse regarding the variable definition and outcome measures examined. Future systematic reviews on this topic should clearly define their definition of AMR, explain the choice of study design, report on the funding of included studies, and assess the impact of risk of bias of individual studies to improve the quality rating.

We summarized all the evidence around community AMR *E. coli* variables available in the literature to date. Variables showed an interrelation between antibiotic use, gut-microbiome interference, and geographical location. Future high-quality research is needed, investigating animal and environmental risk factors related to AMR *E. coli* in humans in the community and collecting data from Africa and Oceania. Additionally, a clear definition for AMR testing consisting of the time between exposure and resistance testing is essential for AMR assessment.

## 4. Materials and Methods

### 4.1. The Search Strategy and Selection Criteria

This umbrella review was conducted according to the guidelines provided in the Joanna Briggs Institute (JBI) Manual for umbrella reviews [33,64,65], and the protocol was registered in PROSPERO (CRD42022316431) [66].

We searched PubMed, EMBASE, and CINAHL for systematic reviews or meta-analyses on the key topics “antibiotic resistance” and “systematic review” (Supplementary Materials). All databases were systematically searched for studies published in English from inception until 25 March 2022, without any geographical restrictions. References and citations of the included studies were screened for additional studies. Citations were screened through the Google Scholar Citation search engine [67] and CoCites, a citation-based search tool [68]. Grey literature was searched via websites of key organizations reporting on antibiotic resistance: the World Health Organization [1], the European Centre for Disease Prevention and Control [69], ReACt group [70], and the Centre for Infectious Disease Research and Policy [23].

The eligibility criteria were based on the Population, Exposure, and Outcome (PEO) design framework [71] (Table 5). We included all systematic reviews investigating AMR *E. coli* in humans of any age living in the community setting or those diagnosed with a community-acquired resistant infection. The community was defined as the space and environment outside of hospitals, nursing homes, or other healthcare institutions. Reviews that included hospital outpatients or those indicating that only hospital-based studies were included when it was clear that the infection was acquired in the community (e.g., diagnosis of infection was within 48 h of admission). Humans working in or affiliated with healthcare facilities were included if they lived in the community, i.e., not admitted to the healthcare facilities. AMR *E. coli* definition included colonization, transmission, acquisition, carriage, or emergence of infection of bacteria resistant to antibiotics or resistant bacterial genes. We included reviews on variables of AMR of the Enterobacterales that included separate results on *E. coli* (Table 5).

We excluded reviews that did not report on the community setting, or that reported on a mix of community and healthcare facilities without presenting the results separately for both settings. Reviews on resistance unrelated to humans, focusing on other specific bacteria than *E. coli*, on nonbacterial resistance and those reporting the prevalence of resistance after variable exposure without any association description between variable and outcome were excluded.

Title and abstract as well as full-text screening were undertaken independently by two reviewers (CCHS and ML) using Covidence (2022) systematic review software www.covidence.org accessed on 13 November 2023, [72]. Any disagreement between reviewers was resolved by discussion. In cases where consensus could not be established, a third or fourth reviewer (LP, KT and/or HR) reviewed the article(s), and discussion took place until consensus was reached between all reviewers involved.

### 4.2. Data Extraction

Two reviewers (CCHS and ML) independently undertook data extraction and methodological quality assessment. Data were extracted using a predefined data extraction tool based on the JBI Data Extraction Form for Systematic and Research Syntheses [33]. The following data (where available) were extracted: review title, author, and year of publication; objectives; type of review; number, type, and region of relevant studies included; population; bacteria investigated; aspect of AMR (emergence, colonization, carriage, infection, or transmission); variables; measures of effect: odds ratio/relative risk and associated 95% confidence intervals; timing between variable exposure and resistance. A third reviewer (LP) independently extracted data from 10% of the included reviews to ensure consistency. All inconsistencies identified during any stage of data extraction were resolved by discussion.

### 4.3. Assessment of Methodological Quality

The methodological quality of the included studies was assessed independently by two reviewers (CCHS and ML) using the “A MeaSurement Tool to Assess Systematic Reviews of both randomized and non-randomized studies” (AMSTAR 2) checklist [33,73]. Any disagreements were resolved by discussion between reviewers or, in uncertainty regarding statistical data, assessment by a statistician (KR).

### 4.4. Data Synthesis

Narrative synthesis was performed for all outcomes and presented in tabular format. Variables were investigated from a One Health perspective as defined by Cars et al. [74]. Additional grouping of variables into subcategories was established after data collection (antibiotic use, health, demographics, travel, pets, farming, and water). No additional contact with the authors of any original papers was sought for clarification or missing data, and one study could contribute to multiple outcomes.

To assess variable importance, we adapted grading scales reported in previous research [75,76] for odds ratios and risk ratios based on effect size scaling by Ferguson et al. [77] (Table 6). Importance was rated by two reviewers (CCHS and LGP), and disagreements were resolved by discussion between reviewers.

## Figures and Tables

**Table 1:** Characteristics of the included reviews.

Study ID	Total Number of Studies That Fulfilled Inclusion Criteria in Umbrella Review (Total Number of Studies in Review)	One Health Category	Population	Continent(s)
Alividza 2018 [34]	11 (19)	Human	Any age group	Asia, Europe, South America
Bakhit 2018 [35]	3 (25)	Human	Any age group	Europe, South America
Bell 2014 [36]	243 (243)	Human	Any age group	Europe, North America, Other ^1^
Bhate 2021 [37]	2 (5)	Human	Aged ≥ 8 years with acne	Europe
Bryce 2016 [38]	6 (34)	Human	Children and adolescents (0–17 years old)	Asia, North America, South America
Bryce 2016 [39]	5 (58)	Human	Children and adolescents (0–17 years old)	Asia, Europe, North America
Butcher 2019 [40]	15 (34)	Human	Any age group	Asia, Europe, South America
Chan 2022 [41]	25 (25)	Human	Children and adolescents (0–18 years old)	Asia
Costelloe 2010 [42]	8 (24)	Human	Any age group	NR
Furuya-Kanamori 2020 [43]	20 (20)	Human	International travelers	Asia, Europe, North America, Oceania
Hackmann 2021 [44]	23 (23)	Animal	Any pet	Asia, Europe, North America, South America
Hassing 2015 [45]	11 (11)	Human	Asymptomatic travelers	Europe, North America, Oceania
Hu 2020 [15]	15 (15)	Human	Healthy population aged 18–65	Africa, Asia, Europe
Karanika 2016 [46]	66 (66)	Human	Healthy individuals	Africa, Asia, Europe, North America, South America, Oceania
Köck 2018 [47]	2 (68)	Animal	Wildlife, food-producing, and companion animals	Asia, Africa
Larramendy 2020 [48]	16 (16)	Human and Environment	Any age group	Africa, Asia, Europe, South America
Lazarus 2015 [49]	34 (34)	Animal	Food-producing animals	Global ^2^
Messina 2020 [50]	4 (30)	Human	Healthy children and adolescents (0–21 years old)	Asia, Europe, North America, Oceania
O’Brien 2019 [51]	3 (19)	Human	Children (1 month to 5 years old)	Africa
Ramblière 2021 [52]	3 (36)	Human	Children (0–15 months old) exposed to HIV and HIV-infected adults	Africa
Truong 2022 [53]	2 (7)	Human	Oral daily tetracycline users	Africa, Asia
Voor In ‘t Holt 2020 [54]	22 (22)	Human	Travelers without infection	Asia, Europe, North America, Oceania
Willems 2020 [55]	4 (26)	Human	Acid suppressant users	Europe

^1^ A total of 61 included studies did not report on the geographical location. ^2^ Included an international study.

**Table 2:** Human health variables of *E. coli* AMR among community-dwelling populations.

Variable	Subcategory	Number of Participants (Number of Studies Investigating Variable)	Magnitude of Association OR (95% CI)	Importance Rating *
Antibiotic use	General antibiotic use	6 studies (NR)	1.51 (1.17–1.94) [15]	+
		1528 (6 studies)	1.58 ** (1.16–2.16) [46]	
		1297 (5 studies)	1.63 ** (1.19–2.24) [46]	
		449 (1 study)	1.8 (1.0–3.1) [48]	
		88 studies (NR)	2.33 (2.19–2.49) [36]	
		NR (5 studies)	2.65 (1.70–4.12) [41]	
		172 (1 study)	3.1 (1.4–6.7) [48]	
		484 (1 study)	4.0 (1.6–10.0) [48]	
		300 (1 study)	4.6 (1.9–11.0) [48]	
		140 (1 study)	5.6 (2.1–14.8) [48]	
	Trimethoprim and β-lactams	179 (2 studies)	3.2 (0.9–10.8) [35]	0
	Beta-Lactam	290 (1 study)	4.5 (1.8–11.0) [48]	+++
		510 (1 study)	4.6 (2.0–10.7) [48]	
	(Fluoro)Quinolone	449 (1 study)	2.1 (0.6–7.3) [48]	+
		200 (1 study)	2.6 (1.3–5.1) [48]	
		140 (1 study)	9.9 (2.2–44.6) [48]	
		290 (1 study)	19.0 (3.3–111.4) [48]	
	Penicillin	7170 (1 study)	0.9 (0.5–1.7) [48]	0
		408 (1 study)	2.7 (1.2–6.3) [48]	
	Cephalosporin	74 (1 study)	1.5 (5.4–85.2) [48]	+
		200 (1 study)	2.2 (1.01–5.0) [48]	
		408 (1 study)	2.2 (1.1–4.5) [48]	
		200 (1 study)	3.9 (1.8–8.5) [48]	
	Macrolides	7170 (1 study)	1.5 (1.1–2.2) [48]	0
	Nitrofurantoin	7170 (1 study)	1.54 (1.1–2.3) [48]	0
	Longer duration of course (>7 days vs. <7 days amoxicillin and trimethoprim)	1521 (2 studies)	1.50 (0.76–2.92) [42]	0
		1521 (2 studies)	2.89 (1.44–5.78) [42]	
	Multiple courses (>3 courses vs. 1 course, trimethoprim, amoxicillin, trimethoprim)	1521 (2 studies)	0.4 (0.12–1.31) [42]	++
		1521 (2 studies)	3.95 (1.06–14.72) [42]	
		1521 (2 studies)	3.62 (1.25–10.48) [42]	
	Mass administration	NR (1 study)	3.64 (2.38–5.78) [51]	+++
		NR (5 studies)	7.8 (3.0–20.2) [52]	
		NR (5 studies)	10.2 (5.9–17.8) [52]	
		NR (5 studies)	17.1 (2.3–127.7) [52]	
	Higher dose (each 200 mg trimethoprim tablet extra and 500 mg instead of 250 mg amoxicillin)	1521 (2 studies)	1.01 (1.01–1.02) [42]	+
		1521 (2 studies)	2.26 (1.13–4.55) [42]	
Comorbidities	Previous/recurrent UTI	7170 (1 study)	1.3 (1.01–1.6) [48]	++
		408 (1 study)	3.4 (1.8–6.7) [48]	
		510 (1 study)	3.8 (1.8–8.1) [48]	
	Previous/recurrent pyelonephritis	300 (1 study)	1.7 (0.7–3.9) [48]	−
	Previous catheterization	408 (1 study)	3.3 (1.7–6.6) [48]	+
	Diarrhea symptoms	5144 (7 studies)	1.53 (1.27–1.84) [15]	0
	Diabetes	300 (1 study)	1.7 (0.8–3.4) [48]	++
		290 (1 study)	3.7 (1.1–12.7) [48]	
		484 (1 study)	3.0 (1.1–8.0) [48]	
	Recurrent acute pyelonephritis and a history of diabetes	300 (1 study)	4.2 (1.3–16.9) [48]	+
	Renal or urological disorder	7170 (1 study)	1.6 (1.0–2.5) [48]	−
		484 (1 study)	3.5 (1.0–11.5) [48]	
	History prostatic disease	510 (1 study)	9.6 (2.1–44.8) [48]	+
	Chronic disease	2323 (3 studies)	0.91 (0.13–6.53) [15]	−
Medication use	Immunosuppressive therapy	7170 (1 study)	1.5 (1.1–2.1) [48]	0
	Corticosteroids	172 (1 study)	24.3 (2.4–246.9) [48]	+
	Acid suppressants	4111 (3 studies)	1.31 (0.11–15.5) [15]	0
		NR (4 studies)	1.41 (1.07–1.87) [55]	
Hospitalization	Previous hospitalization	1379 (5 studies)	1.18 ** (0.78–1.81) [46]	+
		1163 (4 studies)	1.28 ** (0.82–2.03) [46]	
		7170 (1 study)	1.7 (1.3–2.3) [48]	
		172 (1 study)	2.9 (1.3–6.6) [48]	
		7170 (1 study)	3.9 (2.6–5.8) [48]	
		449 (1 study)	3.9 (1.2–12.7) [48]	
	Prior surgery	172 (1 study)	2.8 (1.9–8.0) [48]	0
Diet	Vegetarian	6802 (5 studies)	1.60 (1.0043–2.5587) [15]	0
	Raw milk	226 (1 study)	7.54 (2.41–23.45) [15]	+
	Fish	290 (1 study)	0.6 (0.5–0.9) [48]	0
Sex and age	Older age	300 (1 study)	2.0 (1.02–3.5) [48]	0
	Male sex	NR (9 studies)	0.96 (0.74–1.24) [41]	0
		7170 (1 study)	1.6 (1.2–2.1) [48]	

* Importance rating refers to the statistical significance of a potential variable and/or effect size estimate in relation to *E. coli* AMR; i.e., the amount of studies within the reviews that found statistically significant results (see table in Section 4.4) with +++ very strong association, ++ strong association, + moderate association, 0 weak association and – No association ** Risk ratio (95% CI) instead of odds ratio presented.

**Table 3:** Human living and travel variables of *E. coli* AMR among community-dwelling populations.

Variable	Subcategory	Number of Participants (Number of Studies Investigating Variable)	Magnitude of Association OR (95% CI)	Importance Rating *
Living standards	Lower socioeconomic status	2775 (1 study)	1.33 (1.07–1.75) [34]	+
		2775 (1 study)	2.47 (1.08–5.66) [34]	
	Day-care attendance	NR (6 studies)	1.49 (1.17–1.91) [41]	0
	Living in Northern vs. Southern Europe	7170 (1 study)	0.4 (0.2–0.7) [48]	0
Travel	International travel	1887 (6 studies)	4.06 ** (1.33–2.41) [46]	+++
		834 (1 study)	21 (4.5–97) [48]	
	To Asia	NR (4 studies)	1.78 (0.64–4.98) [15]	++
		NR (12 studies)	14.16 (5.50–36.45) [54]	
		370 (1 study)	30.0 (6.3–147.2) [45]	
	To Africa	NR (3 studies)	0.94 ** (0.14–6.17) [46]	−
	To India	182 (3 studies)	2.4 ** (1.26–4.58) [46]	+
		NR (3 studies)	3.80 (2.23–6.47) [15]	
Health while traveling	Inflammatory bowel disease	5253 (20 studies)	2.09 (1.16–3.77) [43]	0
	Diarrhea	NR (4 studies)	1.65 (1.02–2.68) [15]	+
		5253 (20 studies)	1.69 (1.25–2.30) [43]	
		NR (12 studies)	2.02 (1.45–2.81) [54]	
		430 (1 study)	31.0 (2.7–358.1) [45]	
	Contact with healthcare while traveling	5253 (20 studies)	1.53 (1.09–2.15) [43]	0
	Antibiotic use	5253 (20 studies)	2.38 (1.88–3.00) [43]	+
		NR (12 studies)	2.78 (1.76–4.39) [54]	
		NR (4 studies)	2.81 (1.47–5.36) [15]	
		99 (1 study)	3.0 (1.4–6.7) [45]	
		99 (1 study)	5.0 (1.1–26.2) [45]	
Traveler demographics	Backpackers compared to other travelers	5253 (20 studies)	1.46 (1.20–1.78) [43]	0
	Vegetarian diet	5253 (20 studies)	1.41 (1.01–1.96) [43]	+
		NR (3 studies)	1.92 (1.13–3.26) [15]	
	Diet associated with risk (pastry, meals from stalls, etc.)	NR (12 studies)	1.27 (0.67–2.41) [54]	−
	Street food consumption	NR (2 studies)	0.92 (0.49–1.74) [15]	+
		NR (2 studies)	1.37 (1.08–1.73) [15]	
		NR (2 studies)	2.09 (1.30–3.38) [15]	
	Raw vegetable consumption	NR (2 studies)	0.34 (0.12–0.93) [15]	−
		NR (2 studies)	0.58 (0.33–1.07) [15]	
		NR (2 studies)	2.18 (1.29–3.68) [15]	
Protective measures while traveling	Consuming bottled water	5253 (20 studies)	1.29 (0.50–3.34) [43]	−
	General protective measures (disposable gloves, bottled water, etc.)	NR (12 studies)	0.83 (0.61–1.13) [54]	−
	Meticulous hand hygiene	5253 (20 studies)	1.10 (0.81–1.49) [43]	−
	Probiotics	5253 (20 studies)	1.06 (0.78–1.45) [43]	−

* Importance rating refers to the statistical significance of a potential variable and/or effect size estimate in relation to *E. coli* AMR; i.e., the amount of studies within the reviews that found statistically significant results (see table in Section 4.4) with +++ very strong association, ++ strong association, + moderate association, 0 weak association and – No association ** Risk ratio (95% CI) instead of odds ratio presented.

**Table 4:** Animal and environmental variables of *E. coli* AMR among community-dwelling populations.

Animal	Subcategory	Number of Studies Investigating Variable (Number of Participants)	Magnitude of Association OR (95% CI)	Importance of Rating *
Pets	Pet owner	963 (5 studies)	1.39 ** (0.89–2.18) [44,46]	−
		9403 (12 studies)	1.18 ** (0.83–1.68) [44]	
		5159 (4 studies)	1.15 (0.33–4.06) [15]	
	Dog owner	9403 (12 studies)	0.88 ** (0.56–1.40) [44]	−
	Cat owner	9403 (12 studies)	1.16 ** (0.58–2.34) [44]	−
	Rodent owner	9403 (12 studies)	1.34 ** (0.43–4.18) [44]	−
	Bird owner	9403 (12 studies)	0.91 ** (0.38–2.18) [44]	−
Environment				
Freshwater	Swimming	290 (1 study)	2.1 (1.02–4.3) [48]	0

* Importance rating refers to the statistical significance of a potential variable and/or effect size estimate in relation to *E. coli* AMR; i.e., the amount of studies within the reviews that found statistically significant results (see table in Section 4.4) with 0 weak association and – No association ** Risk ratio (95% CI) instead of odds ratio presented.

**Table 5:** Temporal relationship of variables for *E. coli* AMR among community-dwelling populations.

Variable	Subcategory	Number of Studies Investigating Variable (Number of Participants)	Magnitude of Association OR (95% CI)	Importance of Rating *
Time after antibiotic use	One week	129 (2 studies)	7.1 (4.2–12) [35]	0
	Two weeks	NR (6 studies)	1.08 (0.6–1.96) [38]	+
		NR (1 study)	6.12 (3.18–11.76) [39]	
	One month	NR (6 studies)	1.38 (1.16–1.64) [38]	++
		93 (1 study)	1.8 (0.9–3.6) [35]	
		NR (1 study)	6.20 (2.14–15.96) [39]	
		NR (2 studies)	8.38 (2.84–24.77) [39]	
		1208 (3 studies)	11.21 (7.13–17.63) [51]	
	Two months	14,348 (5 studies)	2.5 (2.1–2.9) [42]	+
		NR (1 study)	5.08 (2.70–9.56) [38]	
	Three months	NR (6 studies)	1.65 (1.36–2.0) [38]	++
		NR (1 study)	3.38 (2.05–5.55) [39]	
		1208 (3 studies)	10.64 (3.79–29.92) [51]	
	Six months	NR (1 study)	3.16 (1.65–6.06) [39]	+++
		1208 (3 studies)	4.76 (1.52–14.90) [51]	
		NR (1 study)	13.23 (7.84–22.31) [39]	
	12 months 11, 51, 54, 59, 60	14,348 (5 studies)	1.33 (1.2–1.5) [42]	+
		NR (1 study)	0.94 (0.57–1.56) [39]	
		10,079 (13 studies)	1.84 (1.35–2.51) [15]	
		NR (1 study)	1.89 (1.04–3.42) [39]	
	Over 12 months	NR (1 study)	0.94 (0.57–1.56) [39]	−
Time after return from travel	Six weeks	290 (1 study)	16.4 (3.4–78.8) [48]	+
	Between six weeks and two years	290 (1 study)	2.2 (1.1–4.3) [48]	0

* Importance rating refers to the statistical significance of a potential variable and/or effect size estimate in relation to *E. coli* AMR; i.e., the amount of studies within the reviews that found statistically significant results (see table in Section 4.4) with +++ very strong association, ++ strong association, + moderate association, 0 weak association and – No association.

**Table 6:** Grading the importance of a variable (adapted from [75,76]).

Category of Importance of Variable	Grading Criteria *
Very strong association: +++	The variable is associated with AMR *E. coli* in all reviews, without exception. More than one study included in the review(s) needed to show a *significant* association and a moderate effect size of OR/RR ** ≥ 3.0.
Strong association: ++	One study, or more than 50% of the studies included in the review(s), showed a *significant* association between the variable and AMR *E. coli* with a moderate effect size of OR/RR ≥ 3.0.
Moderate association: +	The variable is associated with AMR *E. coli* in a single study or in ≤ 50% of studies in the review(s) with a *significant* moderate effect size of OR/RR ≥ 3.0. Or the variable is associated with AMR *E. coli* in >50% of the studies with a small *significant* effect size of OR/RR < 3.0.
Weak association: 0	The variable is associated with AMR *E. coli* in a single study or in ≤50% of studies in the review(s) with a moderate *significant* effect size of OR/RR < 3.0. Or the variable is associated with AMR *E. coli* in >50% of the studies with a moderate *nonsignificant* effect size of OR/RR ≥ 3.0.
No association: −	One study, or more than 50% of the studies included in the review(s), showed an association between the variable and AMR *E. coli* with a small *nonsignificant* effect size of OR/RR < 3.0.

** Variables are placed in the highest importance category that they met the criteria for. * Effect size from [77].

## Data Availability

Study protocol is available on PROSPERO: CRD42022316431. All extracted data is available in Supplementary Table S3 of this manuscript with publication.

## Supplementary Materials

The following supporting information can be downloaded at: https://www.mdpi.com/article/10.3390/ijms242417204/s1.
