# Supplementary material for: One Health Determinants of Escherichia coli Antimicrobial Resistance in Humans in the Community: An Umbrella Review
Source: Int J Mol Sci. 2023 Dec 6;24(24):17204. doi: 10.3390/ijms242417204 (PMC10743193; doi:10.3390/ijms242417204)
Supplement: Supplementary file 1 [file ijms-24-17204-s001.zip › ijms-2745297-supplementary.pdf]

International Journal of
Molecular Sciences

MDPI

Supplementary

Table S1. Reasons for exclusion for excluded literature

Study	Exclusion reason
Allel 2020	Conference abstract
Banik 2022	Unsuitable outcomes
Benie 2020	Conference abstract
Bhate (1) 2020	Conference abstract
Bhate (2) 2020	Conference abstract
Bokhary 2021	Unsuitable outcomes
Cabieses 2019	Conference abstract
Carrillo-Larco 2022	Unsuitable setting
Chatterjee 2018	Unsuitable setting
Chen 2021	Unsuitable setting
Chokshi 2019	Unsuitable study design
Dai 2020	Unsuitable setting
Daniali 2020	Unsuitable study design
De Smalen 2017	Unsuitable outcomes
Desai 2022	Unsuitable study design
Ekwanzala 2018	Unsuitable outcomes
Escher 2021	Unsuitable outcomes
Falagas 2013	Unsuitable setting
Fjalstad 2018	Unsuitable setting
Fouz 2020	Unsuitable population
Hassing 2015	Unsuitable outcomes
Hillier 2002	Unsuitable study design
Hu (1) 2019	Unsuitable setting
Hu (2) 2019	Duplicate
Huijbers 2015	Unsuitable population
Iskandar 2020	Unsuitable study design
Khan 2020	Unsuitable population
Landers 2012	Unsuitable study design
Li 2021	Unsuitable population
Lin 2022	Unsuitable language
Marston 2016	Unsuitable study design
Monger 2021	Unsuitable study design
Muloi 2018	Unsuitable outcomes
Nellums 2018	Unsuitable outcomes
Olaru 2021	Unsuitable setting
Omulo 2015	Unsuitable setting
Osei Sekyere 2021	Unsuitable setting
Patini 2020	Unsuitable setting
Rajagopal 2021	Unsuitable outcomes
Ramblière 2021	Duplicate
Schmiege 2020	Unsuitable outcomes
Sibanda 2011	Unsuitable setting
Smith 2021	Unsuitable pathogen

Tacconelli 2009	Unsuitable study design
Tang 2017	Unsuitable outcomes
Toto-Alzate 2021	Unsuitable study design
Weir 2012	Unsuitable pathogen
Xia 2021	Unsuitable setting
Zainab 2020	Unsuitable outcomes
Zhang 2020	Unsuitable population
Zhu 2020	Unsuitable setting

Table S2. Methodological Quality Assessment Using the A MeaSurement Tool to Assess systematic Reviews 2 (AMSTAR 2)

	**PICO criteria**	**Reported protocol**	**Explain study design**	**Comprehensive search**	**Selection in duplicate**	**Extraction in duplicate**	**List of excluded studies**	**Described in adequate detail**	**Satisfactory technique risk of bias**	**Report on funding included studies**	**Appropriate method for combination results**	**Assess individual impact risk of bias**	**Account for individual risk of bias**	**Explanation and discussion for heterogeneity**	**Adequate investigation publication bias**	**Report on conflicts of interest**	**Overall rating**
**Alividza**	Yes	No	No	No	Yes	No	No	Partial yes	N/A	No	N/A	N/A	No	No	N/A	Yes	Critically low
**Bakhit**	Yes	Partial yes	No	Partial yes	Yes	Yes	No	Partial yes	No	No	No	No	No	No	No	Yes	Critically low
**Bhate**	Yes	Yes	No	No	Yes	No	Yes	Partial yes	Yes	No	N/A	N/A	No	No	No	No	Critically low
**Bryce B**	Yes	Partial yes	No	Yes	Yes	Yes	No	Partial yes	No	No	Yes	No	No	No	No	Yes	Critically low
**Bryce A**	Yes	Partial yes	No	Yes	Yes	Yes	No	Partial yes	No	No	Yes	No	No	Yes	No	Yes	Critically low
**Butcher**	Yes	No	No	No	Yes	Yes	No	Partial yes	No	No	N/A	N/A	No	No	N/A	Yes	Critically low
**Chan**	Yes	No	No	No	No	Yes	No	Partial yes	Yes	No	Yes	No	No	Yes	No	Yes	Critically low
**Costelloe**	Yes	No	No	Yes	Yes	Yes	No	Yes	No	No	Yes	No	No	Yes	No	Yes	Critically low
**Furuya-Kanamori**	Yes	Partial yes	No	Partial yes	Yes	Yes	No	Yes	No	No	No	No	No	No	No	Yes	Critically low
**Hackmann**	Yes	Partial yes	No	No	Yes	Yes	No	Partial yes	Yes	No	Yes	No	No	Yes	No	Yes	Critically low
**Hassing**	Yes	Partial yes	No	Partial yes	Yes	No	No	Yes	Yes	No	N/A	N/A	No	No	N/A	Yes	Critically low
**Hu**	Yes	No	No	No	Yes	Yes	No	Partial yes	No	No	Yes	No	No	No	Yes	Yes	Critically low
**Karanika**	Yes	No	No	No	Yes	Yes	No	No	Yes	No	Yes	No	No	No	Yes	Yes	Critically low
**Köck**	Yes	No	No	No	Yes	Yes	No	No	No	No	N/A	N/A	No	No	N/A	Yes	Critically low
**Larramendy**	Yes	No	No	Partial yes	Yes	Yes	No	Partial yes	No	No	N/A	N/A	No	No	N/A	Yes	Critically low
**Lazarus**	Yes	No	No	Partial yes	Yes	No	No	Partial yes	No	No	N/A	N/A	No	No	N/A	Yes	Critically low

**Messina**	Yes	No	No	No	No	No	No	Partial yes	No	No	N/A	N/A	No	No	N/A	Yes	Critically low
**O'brian**	Yes	No	No	Yes	Yes	Yes	No	Yes	Yes	No	N/A	N/A	No	No	N/A	Yes	Critically low
**Ramblière**	Yes	Partial yes	No	Partial yes	No	No	No	Yes	No	No	N/A	N/A	No	No	N/A	Yes	Critically low
**Truong**	Yes	Yes	No	Partial yes	Yes	Yes	No	Yes	No	No	N/A	N/A	No	No	N/A	Yes	Critically low
**Voor in 't Holt**	Yes	Partial yes	No	Partial yes	Yes	Yes	No	Yes	No	No	No	No	No	No	Yes	Yes	Critically low
**Willems**	Yes	Partial yes	No	Partial yes	Yes	Yes	No	Partial yes	Yes	No	Yes	Yes	Yes	Yes	Yes	Yes	Low

Search strategy for PubMed, EMBASE and CINAHL

PubMed:
((((((Drug Resistance, Microbial/[MeSH Terms]) OR (antimicrobial resistance)) OR (superbug)) OR (super bug)) OR (AMR)) OR (Drug resistan* adj3 Bacteria*)) OR (Drug resistan* adj3 Microbial*)
AND
(((systematic review[ti] OR systematic literature review[ti] OR systematic scoping review[ti] OR systematic narrative review[ti] OR systematic qualitative review[ti] OR systematic evidence review[ti] OR systematic quantitative review[ti] OR systematic meta-review[ti] OR systematic critical review[ti] OR systematic mixed studies review[ti] OR systematic mapping review[ti] OR systematic cochrane review[ti] OR systematic search and review[ti] OR systematic integrative review[ti]) NOT comment[pt] NOT (protocol[ti] OR protocols[ti])) NOT MEDLINE [subset]) OR (Cochrane Database Syst Rev[ta] AND review[pt]) OR systematic review[pt])

EMBASE:
('antibiotic resistance'/exp OR 'antibiotic resistance'/exp OR 'antimicrobial resistance'/exp OR 'super bug'/exp OR 'superbug'/exp OR 'AMR'/exp OR (Drug resistan* adj3 Bacteria*).mp. [mp=title, abstract, heading word, drug trade name, original title, device manufacturer, drug manufacturer, device trade name, keyword heading word, floating subheading word, candidate term word]) OR (Drug resistan* adj3 Microbial*).mp. [mp=title, abstract, heading word, drug trade name, original title, device manufacturer, drug manufacturer, device trade name, keyword heading word, floating subheading word, candidate term word])
AND
Limit to "systematic review" OR "meta analysis"

CINAHL:
#1 MeSH Term "Drug Resistance, Microbial+" OR "antibiotic resistance"
#2 "antimicrobial resistance"
#3 "superbug"
#4 "super bug"
#5 "AMR"
#6 #1 OR #2 OR #3 OR #4 OR #5

Table S3. Details of included reviews as per data extraction form

**Author, year**	**Alividza 2018 [34]**
**Type of review**	Systematic review
**AMR outcome category**	Humans
**Aim of study**	Investigate the association of AMR and poverty by identifying the dimensions of poverty that are potential factors in the acquisition of antimicrobial-resistant organisms in humans.
**AMR definition**	Colonization, Carriage, Infection
**Population and setting**	Different dimensions of poverty in humans in household, community/neighbourhood.
**Overall results**	Level of education, low income, housing conditions, water and sanitation were positively associated with AMR. Among the studies in HICs, positive associations were identified between housing conditions, lack of education and low income, while in turn water and sanitation were positively associated with AMR in LMICs.
**E. coli specific results**	One study did not find any association between low income and *E. coli* resistance. Another study established that community-level indicators of low adult education, skills and training, along with poor living conditions, were two key domains impacting on the prevalence of cefuroxime- and nitrofurantoin-resistant E.coli. In Indonesia, incomplete primary school education was not associated to carriage of resistant E. coli. Similar results have been observed in rural India and the Peruvian Amazon. Researchers in Brazil reported the wide distribution of potentially pathogenic E. coli strains among asymptomatic children of low socioeconomic status. Resistance to either sulphonamides (52%) or cotrimoxazole (35%) was more frequent in cases from the slum (65%) than the control group (16%). In India, the type of household water purification method in two rural villages was associated with carriage of resistant E. coli in primary school children. A UK study used surveillance data of urinary E. coli isolates collected from patients attending primary care services in England with suspected urinary tract infection in 2010–2012. The multilevel logistic regression models estimated that participants' living environment and residency in the most deprived areas was associated with increased odds of antibiotic resistance in the isolates (OR =1·33 [95%CI: 1·07–1·75]–2·47 [95%CI: 1·08–5·66]) for different antibiotics.
**Overall conclusion**	Despite the heterogeneity of the studies, the results suggest an association between a range of dimensions of poverty and antimicrobial-resistant infections across all countries. The variability of results obtained in the E. coli studies exemplifies well the careful equilibrium that societies and health systems have to achieve and sustain between reducing the likelihood of infection with resistant organisms due to lack of material resources versus similar infections resulting from excessive use of antibiotics.
**Author, year**	**Bakhit 2018 [35]**
**Type of review**	Meta-analysis
**AMR outcome category**	Humans

**Aim of study**	To identify and synthesise prospective studies that have examined the occurrence of bacterial resistance in community-based patients who were exposed to antibiotics, and to explore whether resistance decay varies by antibiotic class and bacterium
**AMR definition**	Carriage, Isolation of resistant bacteria
**Population and setting**	Humans (children, symptomatic/asymptomatic patients), in the community exposed to a short antibiotic course
**Overall results**	Antibiotic resistance in either the respiratory or gastrointestinal tracts of people in the community increased immediately after treatment with any of the antibiotics studied. This generally decayed over the next month.
**E. coli specific results**	Trimethoprim and β-lactams exposure: In one RCT (with 64 participants), before antibiotic exposure, the OR of isolating resistance was not significantly different at 0·8 (95% CI 0·3–2·3). Two controlled studies (with 179 participants) compared antibiotic exposure against a group with no exposure. It found that 1 week after antibiotic exposure, the OR of isolating resistant Gram-negative bacteria was 3·2 (95% CI 0·9–10·8). Trimethoprim and trimethoprim-sulfamethoxazole exposure: From two cohort studies (129 participants and 3 different antibiotic exposure groups) the OR of isolating antibiotic-resistant Enterobacteria was 7·1 (95% CI4·2–12) at 1 week. In one study (with 93 participants and 2 different antibiotic exposures), the OR was 1·8 (95% CI 0·9–3·6) at 1 month. In gastrointestinal tract Enterobacteria, there was a significant increase in the odds of isolating trimethoprim-resistant bacteria immediately after exposure to trimethoprim/sulfamethoxazole (OR 4·5, 95% CI 1·8–11·7).
**Overall conclusion**	Resistance generally increased soon after antibiotic use. For some antibiotic classes and bacteria, it partially diminished after 1 and 3 months, but longer-term data are lacking and urgently needed.
**Author, year**	**Bell 2014 [36]**
**Type of review**	Meta-analysis
**AMR outcome category**	Humans
**Aim of study**	To assess the relationship between the antibiotic resistance patterns of bacteria circulating in the community and the consumption of antibiotics in the community
**AMR definition**	Not specified
**Population and setting**	The level of analysis for any given study ranged from the individual patient (or individual bacterial isolate) level to the country (or groups of countries) level. Studies examined either children or adults or both, there were no restrictions on which body sites were sampled for establishing bacterial resistance or how antibiotic consumption was measured, and all bacteria and all antibiotics were considered relevant. Community, out-patient setting.
**Overall results**	There was a positive association between bacterial resistance and antibiotic consumption in the community (OR = 2·33, z = 25·71, p < 0·01, 95% confidence interval 2·19 to 2·49), which means that either increased consumption was associated with increased resistance or decreased consumption was associated with decreased resistance. Five variables were significant independent predictors: both children and adults (z = 3·90 < 0·01), southern Europe (z = 2·25 p < 0·05), B-lactam consumption (z=−3·15 p < 0·01) and quinolone-resistant E coli (QREC) (z = 2·57 p < 0·05).

**E. coli specific results**	First, looking at the results from the binomial tests, we found that only enteric bacteria and streptococcus produced significantly more positive outcomes than negative outcomes ($p < 0{\cdot}01$ for enteric bacteria). Only two correlations were significant for enteric bacteria. Patients who were children ($r = -0{\cdot}28$, $p < 0{\cdot}05$) and quinolone consumption ($r = 0{\cdot}33$, $p < 0{\cdot}01$) were significantly correlated with outcome.
**Overall conclusion**	There is an association between antibiotic consumption and the subsequent development of bacterial resistance at both the individual and community level independently of demographic variables.
**Author, year**	**Bhate 2021 [37]**
**Type of review**	Systematic review
**AMR outcome category**	Humans
**Aim of study**	Investigating published evidence on the association between long-term use of oral antibiotics for acne and subsequent risk of antibiotic treatment failure, infection caused by a resistant organism, or other evidence of AMR
**AMR definition**	Antibiotic treatment failure or any infection caused by a resistant organism, detection without clinical infection
**Population and setting**	Comparator group included people with acne who were not treated with oral antibiotics or the general population in any healthcare setting
**Overall results**	Authors of one study reported pronounced reductions in the numbers of streptococci, enterococci, fusobacteria, and enterobacteria in the colon during the treatment period with oral tetracycline and, in particular, new colonisation with tetracycline-resistant strains was noted. The flora normalised to pre-treatment levels 8 weeks after treatment was stopped. Resistance to tetracycline during treatment was seen in 40% of the staphylococcal and enterococcal isolates from the skin.
**E. coli specific results**	Patients who had received tetracycline for acne and their relatives developed greater numbers of tetracycline Escherichia coli resistant isolates. Conversely, the numbers of erythromycin-resistant E. coli isolates decreased in acne patients receiving an antibiotic for acne but increased in their relatives
**Overall conclusion**	Overall, across all outcomes, low or very low quality of evidence was found supporting long-term oral antibiotics for acne being associated with infectious outcomes or AMR
**Author, year**	**Bryce 2016 [38]**
**Type of review**	Meta-analysis
**AMR outcome category**	Humans
**Aim of study**	Investigate the carriage of faecal E. coli from asymptomatic children resistant to commonly prescribed primary care antibiotics, and quantify the relationship between previous exposure to primary care antibiotics and bacterial resistance
**AMR definition**	Colonization, carriage
**Population and setting**	Children taking antibiotics commonly prescribed in primary care

**Overall results**	Within all antibiotic exposure time periods, the crude odds of resistance were generally greater for children exposed to antibiotics than those unexposed, though exposure at 0–2 weeks was not found to be significantly associated with resistance. The effect sizes are reasonably similar for all time periods, with the pooled OR of resistance rising as the cumulative antibiotic exposure period increases, though CIs do overlap between different time periods. Of studies which reported adjusted ORs, adjusting for family member antibiotic exposure, previous hospitalisation, day care attendance, nappy use, ethnicity and socio-economic status; we compared these results with our crude estimates, for exposure at 0–3 months. The pooled adjusted (OR 1·70, 95 % CI: 1·36–2·12) and crude (OR 1·65, 1·36–2·00) did not differ substantially.
**E. coli specific results**	The routine use of primary care antibiotics could be an important contributor to carriage of resistant E. coli which we showed persists at both 1 and 3 months post-antibiotic prescription and was stronger than compared to 0-2 weeks exposure.
**Overall conclusion**	Resistance to many commonly used primary care antibiotics in faecal E. coli isolates from asymptomatic children ranged from moderate to very high, with resistance being higher in non-OECD countries. Routine antibiotic use is likely to be an important contributor to resistance, which may persist for up to 3 months post-antibiotic treatment. There was no association found between antibiotic exposure within 0–2 weeks and carriage of resistance; this may have been due to insufficient sample size, or the fact that the studies measuring association within this time period were almost 30 years old, whereas the studies measuring associations in other time periods were more recent. Of the six studies included in our meta-analysis, most reported the association between previous antibiotic exposure and resistance within overlapping time periods. This implies that the associations with longer time periods (i.e. 0–3 months) could reflect either long or short-term relationships.
**Author, year**	**Bryce 2016 [39]**
**Type of review**	Meta-analysis
**AMR outcome category**	Humans
**Aim of study**	To systematically review studies investigating the prevalence of antibiotic resistance in urinary tract infections caused by Escherichia coli in children and, when appropriate, to meta-analyse the relation between previous antibiotics prescribed in primary care and resistance
**AMR definition**	Infection
**Population and setting**	Children and young people aged 0-17 presenting with symptoms of urinary tract infection who had provided a urine sample. Primary care, community acquired infections.
**Overall results**	See E. coli specific results
**E. coli specific results**	Studies (5 included in meta-analysis) varied in the combinations of antibiotic resistance and exposure investigated, some reporting resistance and exposure to any antibiotic, while others reported resistance to trimethoprim, co-trimoxazole, or third generation cephalosporins. Effect size show a decline between exposure time periods of 0-1 month (pooled odds ratio 8.38, 95% confidence interval 2·84 to 24·77) and 0-3 months (3·38, 2·05 to 5·55), then an increase at 0-6 months (13·23, 7·84 to 22·31). There is some evidence (1 study, multiple urinary isolates per child) of decreasing resistance for increasing time from antibiotic prescribing (trimethoprim) with follow-up longer than 365 days: odds ratio, CI (days after exposure): 6·12, 3·18-11·76 (1-14); 6·20, 2·14-15·96 (15-28); 5·08, 2·70-9·56 (29-84); 3·16, 1·65-6·06 (85-168); 1·89, 1·04-3·42 (169-365); 0·94, 0·57-1·56 (>365). A meta-regression analysis on the

	crude odds ratios calculated from the paper by Duffy and colleagues, showed a β coefficient of −0·4 (95% confidence interval −0·61 to −0·19), indicating an important time trend.
**Overall conclusion**	Prescribing of antibiotics to individual children in primary care is an important contributor to bacterial resistance, which can persist for up to six months after prescription. Consistent with our previous review, we found some evidence from Duffy and colleagues of decreasing resistance for increasing time from antibiotic prescribing.
**Author, year**	**Butcher 2019 [40]**
**Type of review**	Systematic review
**AMR outcome category**	Humans
**Aim of study**	Characterize the current body of knowledge regarding risk factors for community-acquired urinary tract infection (CA-UTI) caused by extended-spectrumβ-lactamase-producing (ESBL) uropathogenic Escherichia coli (UPEC). Purpose is to identify major knowledge gaps, suggest potential areas for improved public health intervention, and propose future research directions
**AMR definition**	Infection
**Population and setting**	The first search focused on risk factors for CA-UTI in both adults and paediatric patients caused by ESBL-producing UPEC. We then undertake a more detailed examination of potential risk factors for transmission of ESBL-producing UPEC by reviewing the current body of knowledge that addresses the role of food and environmental sources for ESBL-producing ExPECs that cause CA-UTI. Study settings included hospital inpatient, community clinic, and hospital outpatient services.
**Overall results**	See *E. coli* specific results
**E. coli specific results**	Of the 103 risk factors assessed in the 15 studies, 8 were deemed commonly assessed by this review. The most frequent risk factors assessed were: previous hospitalization (11), antibiotic use within the past 3 months (9), male sex (9), pre-existing condition of type II diabetes (5), previous UTI (6), recurrent UTI (5), previous catheterization (5), and urinary tract abnormality (4). Statistically significant associations between commonly assessed potential risk factors and CA-UTI caused by ESBL-producing E.coli were found to vary between studies. For example, seven (78%) of nine studies found a statistically significant association between antibiotic use in the past 3 months. Male sex was found to be a significant risk factor for ESBL-producing E. coli infection in five (56%) of nine studies. Previous hospitalization was the most common risk factor examined across studies and was found to be a significant potential risk factor in 8 (73%) of 11 studies. Previous UTI caused by any organism was also found to be significantly associated with CA-UTI caused by ESBL-producing E. coli, reported in six of six studies. Recurrent UTI, defined as 3 episodes of UTI in the previous 12 months, was found to be a significant potential risk factor in 5 of 5 studies. The potential risk factors with the highest percentage of patients who were infected with ESBL-producing UPEC were previous UTI(79%) and previous catheterization (60%).
**Overall conclusion**	The risk factors we found for CA-UTI caused by ESBL-producing UPEC reported in the reviewed articles include broad categories that may not be specifically related to UPEC organisms that produce ESBL. Factors such as previous UTI episodes and recurrent UTI may represent risk factors for any drug-resistant UTI and not necessarily UTI caused by ESBL-producing UPEC. Such observations may result from our current lack of precise understanding of mode of transmission of CA-UTI. Furthermore, this review found compelling evidence of the presence of ESBL-producing pandemic

	UPEC lineages that have been implicated in some human cases of CA-UTI in food animals, companion animals, and other environmental sources. These results may suggest that there are in fact point sources of human exposure to these pathogens.
**Author, year**	**Chan 2022 [41]**
**Type of review**	Meta-analysis
**AMR outcome category**	Humans
**Aim of study**	As carriage of AMR S. aureus, S. pneumoniae, Escherichia coli and Klebsiella pneumoniae are associated with significant morbidity and mortality in infected individuals, including children, this study aimed to systematically review the current literature on the risk factors associated with carriage of these four of the most commonly carried bacteria in the community paediatric population in Asia-Pacific regions.
**AMR definition**	Carriage
**Population and setting**	Healthy paediatric population (children 0-18 years old) of Asia-Pacific regions in the community
**Overall results**	Of the 16 studies eligible for meta-analysis, risk factors such as day-care attendance and antibiotic use within the past 3 months have been associated with increased carriage of AMR bacteria of interest while sex was not associated with increased AMR bacterial carriage. Nine studies reported sex can be a risk factor for AMR bacterial carriage while two studies reported male sex is a risk factor and six studies reported the opposite findings. The pooled OR for male sex is 0·96 (95% CI, 0·74–1·24). The heterogeneity of the meta-analysis was moderate (I2= 68%). The pooled results in our meta-analysis of the six studies support an association between day-care attendance and risk for AMR bacterial carriage [OR 1·49 (95% CI 1·17–1·91, I2=57%)]. The pooled results in our meta-analysis of five studies reported that antibiotic use within the past 3 months among young children is a risk factor for carriage of AMR bacteria (penicillin non-susceptible S. pneumoniae, MRSA and ESBL-producing Enterobacteriaceae). The pooled estimate [OR 2·65 (95% CI 1·70–4·12)] shows that the results are significant. The heterogeneity was moderate (I2=31%).
**E. coli specific results**	3 studies investigating E. coli specific resistance. One cross sectional study from Laos, investigating stools from 397 healthy children ≤6 years of age in preschool childcare facility in March–June 2011. 92 children in 397 children, 78 E. coli colonies in 100 colonies. ESBL E. coli 92/397 X 78/100 = 18.1% prevalence. Outcome of interest was antibiotics use in the last 3 months. Results showed that antibiotic use within the past 3 months among young children is a risk factor for carriage of E. coli resistant isolates. Another cross sectional study from India investigated stool samples from 529 children aged 3-14 years old in villages. ESBL E. coli prevalence was 9%. Outcomes of interest were female sex, antibiotics use in the past 3 months and family type. They found female sex as a risk factor for carriage. The last study was a cross sectional from Vietnam in which they investigated faecal samples from 818 preschool children aged 6-60 months between March and June 2007. 60% of E. coli was resistant to 3 or more antibiotics. Outcomes of interest were geographic location of residency-living in highland area. They reported geographic location as a significant risk for AMR carriage.
**Overall conclusion**	The findings of this meta-analysis and narrative review of epidemiological studies support that there are several significant risk factors associated with carriage of AMR bacteria in the Asian-Pacific paediatric population
**Author, year**	**Costelloe 2010 [42]**
**Type of review**	Meta-analysis

**AMR outcome category**	Humans
**Aim of study**	Investigating the effect of antimicrobial use on emergence of resistance in the community. And quantifying the strength and duration of any association as well as identifying which antibiotics were most and least likely to cause resistance.
**AMR definition**	Not specified
**Population and setting**	Adults and children. Primary/ambulatory care (all studies were based in countries were antibiotics are available by prescription only).
**Overall results**	In comparisons of different antibiotics in the same antibiotic classes for effects on resistance, we found no evidence that one class led to reduced resistance compared with another. Effects were strongest in the month directly after prescription but were detectable for up to 12 months. Moreover, we found evidence of a dose-response relation for two commonly prescribed first line antibiotics in primary care, amoxicillin and trimethoprim (higher dose is higher odds of resistance).<br><br>Hillier et al found greater rates of resistance associated with higher doses of amoxicillin (OR 2·3, 95% CI 1·1 to 4·6) and longer courses of trimethoprim (2·9, 1·4 to 5·8), but no differences associated with different course durations for amoxicillin (1·5, 0·7 to 2·9). They also found associations between number of courses of amoxicillin (three or more v one; OR 3·9, 95% CI 1·0 to 14·7) and trimethoprim (three or more v one; 3·6, 1·2 to 10·5) and resistance. The Hay study showed no differences in resistance rates with differing numbers of antibiotic courses, but did find an increase of 1% in the odds of resistance for each 200 mg trimethoprim tablet prescribed (OR 1·01, 95% CI 1·01 to 1·02). The same type of association was not found for increasing numbers of 500 mg amoxicillin tablets prescribed.
**E. coli specific results**	Resistance for urinary bacteria (7 studies included in meta-analysis): Across all periods the odds of resistance were greater in patients exposed to these antibiotics than in those who were unexposed and that the strongest association was at 0-1 months (pooled odds of resistance among unexposed participants was 0·44 at 0-1 month), with reduced association at subsequent time points, and a small but important residual association within 12 months. The βcoefficient for each month increase in the exposure period was −0·33 (95% CI−0·49 to−0·17, P<0·001) from the meta-regression showing a clear time trend.
**Overall conclusion**	Individuals prescribed an antibiotic in primary care for a respiratory or urinary infection develop bacterial resistance to that antibiotic.
**Author, year**	**Furuya-Kanamori 2020 [43]**
**Type of review**	Meta-analysis
**AMR outcome category**	Humans
**Aim of study**	Quantify the risk factors and interventions for reducing the risk of multidrug-resistant Enterobacterales (MRE) acquisition among international travellers
**AMR definition**	Colonization; Acquisition; Selection

**Population and setting**	International travellers
**Overall results**	See E. coli specific results
**E. coli specific results**	The vast majority of the 2276 MRE isolates was E. coli (92·0%; 95% CI 84·9–97·3%). MRE detection was not associated with sex, age or duration of travel, but it was dependent on the travel destination. It was found that inflammatory bowel disease (OR 2·1; 95% CI 1·2–3·8) was a significant risk factor for MRE colonization. While overseas, use of antibiotics (OR 2·4; 95% CI 1·9–3·0) was the strongest risk factor. Other factors associated with MRE colonization were experiencing traveller's diarrhoea (OR 1·7; 95% CI 1·3–2·3) and having contact with the healthcare system either as inpatients or outpatients (OR 1·5; 95% CI 1·1–2·2). Antimalarial prophylaxis (OR 1·0; 95% CI 0·8–1·4) was not associated with MRE colonization, but it should be noted that commonly used antimalarial medications include doxycycline (abroad spectrum antibiotic) as well as other medications (e.g. mefloquine, atovaquone/proguanil, chloroquine) that would not be expected to have an effect on selection of AMR Enterobacterales. With regards to food exposure, having a vegetarian diet (OR 1·4; 95% CI 1·0–2·0) was found to increase the odds of acquiring MRE compared to other diets. Oral cholera vaccine (OR 1·6; 95% CI 1·0–2·5) was found to increase the odds of MRE colonization, possible due to confounding by indication. Backpackers had a 50% (OR 1·5; 95% CI 1·2–1·8) increased odds of acquiring MRE compared to other types of travellers.
**Overall conclusion**	International travel is an important driver for MRE spread worldwide. South Asia as a travel destination is a major risk factor for MRE acquisition. The highest proportions of MRE detection were observed from travellers returning from South Asia, Northern Africa, Asia, sub-Saharan Africa and South and Central America. Additional risk factors for individuals to become colonized include inflammatory bowel disease, use of antibiotics, traveller's diarrhoea, contact with the healthcare system, having a vegetarian diet and backpacking
**Author, year**	**Hackmann 2022 [44]**
**Type of review**	Meta-analysis
**AMR outcome category**	Animals
**Aim of study**	Assessing contact to pets as a risk factor for acquisition of MRSA, VRE and MDR Gram-negatives [namely third-generation cephalosporin-resistant Enterobacterales (3GCRE) and carbapenem-resistant Enterobacterales (CRE)
**AMR definition**	Carriage
**Population and setting**	Any pet that is in contact with humans. Any setting thus hospital, community etc. is considered
**Overall results**	See E. coli specific results
**E. coli specific results**	Meta-analysis was divided into subgroups based on pet species. Four out of 13 publications reported data separately for different animal species. The respective datasets were therefore included in the subgroup analyses, but spared the summarizing analysis to prevent over representation of studies considered multiple times in the meta-analysis. Overall, the meta-analyses consisted of 13 studies and included 10 820 participants (range 48–4177). All studies investigated infection or colonization with 3GCRE. Eleven out of 13 publications focused on ESBL-E, with E. coli being the most represented pathogen. None of the publications reported CRE colonization. Studied pet species comprised dogs, cats, rodents, birds and reptiles/amphibians. None of

	the meta-analyses showed a significantly higher risk for 3GCRE colonization in owners of different pet species compared with non-pet owners (RR 1·18, 95% CI 0·83–1·68 for pet owners in general, RR 0·88, 95% CI 0·56–1·40 for dog owners, RR 1·16, 95% CI 0·58–2·34 for cat owners, RR 1·34, 95% CI 0·43–4·18 for rodent owners, RR 0·91, 95% CI 0·38–2·18 for bird owners). Heterogeneity was low-to-moderate in the subgroups of pet owners in general, dog owners and bird owners (I2= 0%–59%). The subgroups of cat owners and rodent owners showed critical heterogeneity (I2= 74%–83%). Since these subgroups only comprise three to four publications, no further subgroup analysis was performed. Therefore, we performed a sensitivity analysis. The publications varied in methodical and clinical characteristics, but changes in study selection, effect measure or a change to fixed-effect model did not show significant improvements in heterogeneity. To prevent limitation of the informative content of this analysis, we proceeded to include these subgroups, but results have to be discussed critically. Studies were of high (5/13) or moderate (8/13). The number of included studies was too small for each subgroup to estimate the reporting bias based on a funnel plot, but in total, the findings appear to be distributed symmetrically around the pooled RR.
**Overall conclusion**	Data on pet ownership as a risk factor for 3GCRE and CRE colonization are still too scarce to come to a definite answer. However, several studies confirmed that transmission is possible and matching resistance genes can be found in pets and owners. Bacteria from pets can harbour resistance genes originally found in bacteria from livestock and, therefore, act as vectors between livestock and humans, but also act as a reservoir for human strains of 3GCRE.
**Author, year**	**Hassing 2015 [45]**
**Type of review**	Systematic review
**AMR outcome category**	Humans
**Aim of study**	To determine the effect of international travel on the risk of post-travel faecal carriage of multidrug-resistant Enterobacteriaceae (MRE). A secondary objective was to determine risk factors for acquisition of drug resistance.
**AMR definition**	Colonization, acquisition, carriage
**Population and setting**	Asymptomatic travellers
**Overall results**	See E. coli specific results
**E. coli specific results**	Faecal carriage of MRE varied from 1 to 12% before travel and acquisition of MRE from 21% to 51%. In the study by Kuenzli et al. on travellers to the Indian subcontinent only, a much higher MRE acquisition rate of 69% was demonstrated. The risk of acquisition of MRE varied with the geographical region. Travel to southern Asia posed the highest risk (range: 29–88%), followed by other Asian countries (18–67%) and Northern Africa (range: 31–57%). Acquisition of MRE after travelling to sub-Saharan Africa (range: 0–49%) or South and Central America (range: 0–33%) was less frequent, and three studies did not observe any acquisition of MRE after travel to South or Central America. Acquisition of MRE after travel to North America, Europe and Oceania was rare. Besides travel destinations, other risk factors for acquiring MRE were age, use of antibiotics during travel (beta-lactam use) and gastroenteritis or other gastrointestinal symptoms. The study of Kantele et al., designed to study these risk factors as primary outcome, showed that travel diarrhoea (adjusted odds ratio (AOR) = 31·0; 95% confidence interval (CI): 2·7–358·1)) and antibiotic therapy for travel diarrhoea (AOR = 3·0; 95% CI: 1·4–6·7) proved to be the most important risk factors for acquiring MRE. In the study of Kuenzli et al. in which only travellers to southern Asia were

	included, risk factors for MRE acquisition were length of stay, visit to family or friend and consumption of ice cream or pastry. Angelin et al. found a significant association for travel to the South-East Asia region (OR = 30; 95% CI: 6·3–147·2), and antibiotic treatment during travel (OR = 5; 95% CI: 1·1–26·2), but found no association with travellers' diarrhoea or patient-related healthcare work. Resistance of post-travel MRE isolates to various antibiotics was determined in nine studies. In the study by Wintersdorff et al., a PCR-based approach was used, therefore it was not possible to determine which microorganism carried the resistance genes. The resistance data to other antibiotic drugs in the study by Kennedy et al. were not part of the publication, but were provided on request. Antimicrobial resistance was high for ciprofloxacin, varying from 31% to 57%, and for cotrimoxazole, varying from 49% to 86%. Aminoglycoside resistance was high for gentamicin (range: 17–50%) and tobramycin (range: 18–59%) and low for amikacin (range: 2–5%). Carbapenemase-producing Enterobacteriaceae were observed in four travellers who had all visited India (in the study by Ruppé et al., two OXA-181 and one New Delhi metallo-beta-lactamase 1 (NDM-1), and in the study by Kuenzli et al., one NDM-1 but this strain was not included in the resistance results). Resistance to nitrofurantoin, colistin and fosfomycin was only analysed in some of the studies. Five studies analysed MRE carriage six months after travel, and the persistence rate of acquired MRE after six months was 6–24% of travellers. Ruppé et al. analysed MRE carriage one, two, three, six and twelve months after travel, showing persistence of carriage of an acquired MRE in 34, 19, 10, 5 and 2%, respectively. Travellers to Asia showed longer carriage of MRE compared with other travel destinations. Carriage of multidrug-resistant E. coli had a lower risk for prolonged carriage than other multidrug-resistant species. No other risk factors were found for prolonged carriage of MRE. Eight travellers in this study reported an episode of urinary tract infection after their return, but no microbiological data were available. In the study by Tängdén et al., five of 21 travellers remained carriers of MRE after six months. However, none of these participants reported clinical infections. In the study of Kennedy et al., one person developed a urinary tract infection with a travel-related organism. Kantele et al. performed a one-year laboratory-based follow-up and did not find any clinical samples with MRE. Only one study screened household contacts for MRE after return of the index traveller. Household contacts were defined as persons who shared the same household with a participant on a regular basis. Two of 11 contacts were found MRE-positive. Both carried a different ESBL-producing E. coli based on multilocus sequence typing (MLST) than the associated traveller.
**Overall conclusion**	International travel is a major risk factor for acquisition of MRE. This risk is particularly high after travelling to (southern) Asia and in persons with travel-related diarrhoea and antibiotic use. Carriage of MRE-positive isolates is also associated with a high risk of resistance to ciprofloxacin, cotrimoxazole and aminoglycosides.
**Author, year**	**Hu 2020 [15]**
**Type of review**	Meta-analysis
**AMR outcome category**	Humans
**Aim of study**	Investigate risk factors associated with intestinal carriage of drug-resistant commensal E. coli in the recent five years and identify risk factors related to food in healthy adult population
**AMR definition**	Carriage
**Population and setting**	Eight studies sampled volunteers from healthy general population that were registered to a hospital system. Five were cohort studies of healthy travellers that compared the prevalence of drug-resistant Enterobacteriaceae or E.coli before and after the travel. Two studies surveyed pig farmers

**Overall results**	See *E. coli* results
**E. coli specific results**	The results showed that antimicrobial use within the previous 12 months (OR 2·81 [95% CI 1·47-5·36]), diarrhoea symptoms (OR 1·65 [95% CI 1·02-2·68]), vegetarian diet (OR 1·92[95% CI 1·13-3·26]), and travelling to India (OR 3·80 [95% CI 2·23-6·47]) remained significant risk factors of faecal carriage of drug-resistant *E. coli* among travellers.<br><br>Among general population adults, antimicrobial use within the previous 12 months (Pooled OR = 1·51 [95% CI1·17–1·94]), diarrhoea symptoms (OR 1·53 [95% CI 1·27-1·84]), and travelling to Southeast Asia (OR 1·67 [95% CI1·02-2·73]) were significant risk factors.<br><br>Six of 15 studies reported risk factors related to food. Five studies assessed the risk among vegetarians. As stated above, pooled OR showed significant association with being a vegetarian (OR 1·60 [95% CI 1·00-2·56]); two studies reported significant association, one with unadjusted OR, and another with adjusted OR. Four studies reported potential food-associated risk factors other than being a vegetarian. One study reported exposure to raw milk as significant risk factor for acquiring multi-drug-resistant E.coli (OR 7·54 [95% CI 2·41-23·45]). Two studies reported the effect of eating street food during travel. One of them was reported as significant risk (OR 2·09 [95% CI 1·30-3·38] for daily consumption; OR was 1·37 [95% CI 1·08-1·73] for occasional consumption during travel). Another study did not find significant association (OR 0·92 [95% CI 0·49-1·74]). Two studies assessed the effect of raw vegetable consumption on the faecal carriage of drug-resistant E. coli. One of them reported that raw vegetable consumption during a trip to Southeast Asia significantly increased the risk of intestinal carriage of drug-resistant Enterobacteriaceae (OR 2·18 [95% CI 1·29-3·68]), while exposure to raw vegetable in South Asia significantly decreased the risk (OR 0·34 [95% CI 0·12-0·93]). The other study did not find any significant association (OR 0·58 [95% CI 0·33-1·07])
**Overall conclusion**	We found five significant risk factors associated with intestinal carriage of drug-resistant E.coli, antimicrobial use, diarrhoea, vegetarian diet, travel to India, and travel to Southeast Asia.
**Author, year**	**Karanika 2016 [46]**
**Type of review**	Meta-analysis
**AMR outcome category**	Humans
**Aim of study**	Estimate the prevalence of Extended-spectrum beta-lactamases (ESBL) class A (mainly represented by cefotaximase [CTX-M], temoneira [TEM], and sulfhydryl variable [SHV]) colonization among healthy patients and assess the factors that are associated with the colonization status
**AMR definition**	Colonization, carriage
**Population specifics**	Healthy individuals (explicitly characterized as such or reported as asymptomatic and were recruited from the community voluntarily for a research purpose without seeking medical advice for any health problem)
**Setting specifics**	Community
**Overall results**	See E. coli specific results

**E. coli specific results**	Based on 6 studies, providing data on 1528 individuals and their antibiotic use within 12 months, individuals treated with antibiotics were significantly more likely to be colonized with ESBL (RR = 1·58; 95% CI, 1·16, 2·16; Q = 3·27, PQ = 0·66; ET = −1·10, PET = 0·08). Also, 5 of the studies reported antibiotic use during the previous 4 months for 1297 individuals, and these individuals were 63% more likely to be colonized with ESBL compared with those not treated with antibiotics (RR = 1·63; 95% CI, 1·19, 2·24; Q = 2·32, PQ = 0·68; ET = −0·95, PET = 0·20). Further data on the stratification per antimicrobial class were not provided in individual studies. Thus, no correlation between a specific antibiotic class and the risk of colonization could be made. Based on 6 studies that provided data on 1887 individuals, those who travelled internationally were >4 times more likely to be colonized with ESBL (RR = 4·06; 95% CI, 1·33, 12·41; Q = 50·96, PQ = 0·00; ET = 0·68, PET = 0·90; Regarding travel destination, based on 3 studies on 182 individuals, those who travelled to India were 240% more likely to be colonized with ESBL compared with those who travelled to any other destination (RR = 2·4; 95% CI, 1·26, 4·58; Q = 18·44, PQ = 0·00; ET = 5·32, PET = 0·28). Travel to Africa was tested as a potential high-risk destination but did not increase the risk for ESBL colonization (RR = 0·94; 95% CI, 0·14, 6·17; Q = 13·44, PQ = 0·00; ET = 0·71, PET = 0·88). No significant association was found between colonization and either lifetime hospitalization (5 studies on 1379 individuals; RR = 1·18; 95% CI, 0·78, 1·81; Q = 2·28, PQ = 0·67; ET = 0·09, PET = 0·91) or hospitalization within the previous year (4 studies on 1163 individuals; RR = 1·28; 95% CI, 0·82, 2·03; Q = 1·44, PQ = 0·70; ET = 0·30, PET = 0·70). In addition, no significant association between animal contact and the risk of colonization was found based on 5 studies on 963 individuals (RR = 1·39; 95% CI, 0·89, 2·18; Q = 4·73, PQ = 0·32; ET = 1·74, PET = 0·14). In multivariate meta-regression analysis, among the preselected assessed factors/covariates, the prevalence of ESBL colonization was significantly affected by the geographical distribution, showing that the West Pacific and Southeast Asia demonstrated the strongest positive association with ESBL colonization rate. Predictably, the publication of new guidelines in 2010 was also a significant factor that affected our estimated prevalence. Overall, 49.80% (adjusted R-sq) of the between-study variance was found to be justified by the listed covariates.
**Overall conclusion**	Healthy individuals are an important reservoir for ESBLs and the colonization rate appears to be increasing over time.
**Author, year**	**Köck 2018 [47]**
**Type of review**	Systematic review
**AMR outcome category**	Animals
**Aim of study**	Evaluate the occurrence and prevalence of Carbapenem Resistant Enterobacteriaceae in animal species as reported in the literature thus far.
**AMR definition**	Transmission
**Population and setting**	Enterobacteriaceae in samples obtained from livestock (pigs, poultry, cattle), farm environments, slaughterhouses, food items derived from these animals, as well as seafood, undomesticated animals (wildlife), companion animals (dogs, cats, horses), or humans directly exposed to all therefore mentioned animals
**Overall results**	We found that CRE occur globally in livestock, pets, wildlife, and seafood
**E. coli specific results**	Among the samples from one Chinese commercial chicken farm, (Wang et al.) tested six anal/faecal specimens from farmers, of which three (50·0%) were positive forblaNDM-5-positive CR E. coli. They found that blaNDM genes were located on different plasmids of replicon types i.) HI2, Y, FrepB,

	A/C; ii.) FIB; and iii.) FIC, FrepB, I1, HI2, FIB. Transferability was confirmed in conjugation experiments. They also compared the E. coli isolates from farmers to >150 E. coli isolates from the respective farm and its environment using whole-genome sequencing followed by typing of core genome single-nucleotide polymorphisms (cgSNP). This analysis revealed that two of the farmers' isolates shared MLST types (ST10, ST746) with isolates from local dogs, flies, and chickens and clustered closely together, suggesting transmission on the farms. Moreover, Wang et al. demonstrated clonal commonality between blaNDM-positive E. coli strains from chicken farms, slaughterhouses, supermarkets, and humans, belonging to MLST types ST10 andST156 using cgSNP-based phylogenetic analysis. Hamza et al. collected faecal samples from 30 workers and 19 veterinarians in five poultry farms in Egypt, of whom four workers (13·3%) and one veterinarian (5·3%) carried CRK. pneumoniae. PCRs targeting resistance genes showed that all five isolates harboured a combination ofblaOXA, blaKPC, andblaNDM genes, which corresponded to findings for K. pneumoniae isolates from poultry on these farms. However, comparisons of clones or plasmids were not performed in this study.
**Overall conclusion**	Two studies where transmission of CRE between animals and exposed humans was assessed. These examples demonstrated that direct anthropozoonotic or zooanthroponotic transmission is possible for CRE. However, for estimating the public health relevance of this transmission, this review underscores the necessity for more data about the transmission probability
**Author, year**	**Larramendy 2020 [48]**
**Type of review**	Systematic review
**AMR outcome category**	Humans
**Aim of study**	Identify risk factors of carrying ESBL-producing E. coli among patients with UTI in order to optimize their management in the community.
**AMR definition**	Carriage
**Population and setting**	Adult or paediatric populations without age restrictions in the community (i.e., outside hospitals or medium- and long- stay centres)
**Overall results**	Previous use of antibiotics, hospitalization in the previous months, and history of UTIs were known risk factors. Travelling to endemic areas seems also an important risk factor (OR: 16·4 [3·4–78·8]) and is present for several months after travelling, with it decreases over time.
**E. coli specific results**	Previous antibiotic intake was the most frequently identified risk factor for UTI due to ESBL-producing E. coli (n=12 studies) and was strongly associated with UTI occurrence in most of these studies (OR >4 in 8 of these 12 studies). Prior hospitalization was identified as a risk factor in three good-quality studies (NOS score: 7 to 9). The OR values ranged between 1·7 (95% CI: 1·3–2·3) and 3·9 (95% CI: 1·2–12·7), depending on the number of prior hospitalizations and time to infection. UTI history was identified as a risk factor in three studies. The ORs ranged between 1·3 (95% CI: 1·0–1·6) and 3·8 (95% CI: 1·8–8·1), but the definition of "UTI history" was heterogeneous: UTI during the previous year, ≥3 UTI episodes during the previous year, and recurrent acute pyelonephritis. Diabetes was a moderated risk factor for UTI due to ESBL-producing E. Coli (OR: 3·7 (95% CI: 1·1–12·7)) and a synergistic effect of diabetes with recurrent acute pyelonephritis was reported (OR: 4·2, 95% CI: 1·3–16·9). Catheter-related UTI (OR: 3·3, 95% CI: 1·7–6·6), surgery 3–12 months before infection (OR: 2·7, 95% CI: 1·9–8·0), use of immunosuppressive treatments (OR: 1·5; 95% CI: 1·1–2·1), and chronic corticosteroid treatments (OR: 24·3; 95% CI: 2·4–246·9) were identified as risk factors for UTI due to ESBL-producing E. coli. Care-related infection risk factors were also investigated as a cluster in two different studies and included the following risk factors: living in a nursing home, hospitalization in

	the previous 3 months, antibiotic use in previous 3 months, having received haemodialysis, intravenous treatment or wound care at home in previous 30 days. Age over 55 (OR: 2·0, 95% CI: 1·0–3·5) and male sex (OR: 1·6, 95% CI: 1·2–2·1) also have been reported as risk factors. Lastly, travelling abroad (Asia, Middle East, Africa)(OR: 21; 95% CI: 4·5–97) in previous 6 weeks and swimming in freshwater (OR: 2·1; 95% CI: 1·0–4·2) were identified.
**Overall conclusion**	The most relevant risk factors for CA-UTI due to ESBL-EC identified were prior use of antibiotics (odds ratio (OR) from 2·2 to 21·4), previous hospitalization (OR: 1·7 to 3·9), and UTI history (OR: 1·3 to 3·8). Two risk factors were related to environmental contamination: travelling abroad, and swimming in freshwater.
**Author, year**	**Lazarus 2015 [49]**
**Type of review**	Systematic review
**AMR outcome category**	Animals
**Aim of study**	Investigate all available published evidence that supported, or did not support, the hypothesis that Food producing animals (FPAs) are a source of extra intestinal Expanded-Spectrum Cephalosporin-resistant-Escherichia Coli (ESCR-EC) infection in humans
**AMR definition**	Transmission
**Population specifics**	Food producing animals and humans
**Setting specifics**	Community
**Overall results**	Poultry in the Netherlands have been implicated as a likely source of human infections. A variety of FPA and geo-graphical regions have also been investigated, but the evidence is mixed. Whole bacterium and MGE-mediated mechanisms of transmission may both play a role.
**E. coli specific results**	Six published articles provide data to support the hypothesis of WBT of ESCR-EC between poultry and extra intestinal human sites. Thirteen studies provide data to support the hypothesis of MGE-mediated transmission of ESCR between animal bacteria and E. coli causing human extra intestinal infections. Seventeen studies did not demonstrate direct WBT of ESCR-EC in North America, Europe, and Asia. Two studies did not demonstrate MGE-mediated transmission of ESCR genes between E. coli from FPAs and human sources. Geographically and temporally matched isolates between 2006 and 2010, found a similar distribution of ESBL genes, and, using multilocus sequence typing(MLST), found that animal- and human-sourced bacteria clustered in identical clonal complexes (in poultry flocks, retail chicken and human rectal and blood culture isolates).
**Overall conclusion**	There is evidence that a proportion of human extra intestinal ESCR-EC infections originate from FPAs. Poultry appears (specifically in the Netherlands) to be a more likely source than other FPAs based on current evidence. Transmission of whole ESCR-EC and mobile ESCR genetic elements from poultry to humans probably occurs, but the specific parameters surrounding this, including the magnitude and geographical extent of the problem, remain inadequately understood.
**Author, year**	**Messina 2020 [50]**
**Type of review**	Systematic review

**AMR outcome category**	Humans
**Aim of study**	Identify factors that influence the carriage of AR bacteria in healthy children in the community.
**AMR definition**	Carriage
**Population and setting**	healthy children in the community
**Overall results**	The majority of risk factors were assessed in 2 or fewer studies per bacteria. Recent antibiotic consumption was associated with carriage of resistant respiratory bacteria (S. pneumoniae, H. influenzae); however, it was not consistently associated with carriage of AR bacteria in skin or stool (S. aureus and E. coli). For AR S. aureus, transmission within households appeared to have a greater impact than individual antibiotic use
**E. coli specific results**	Risk factors associated with carriage of AR E. coli were investigated in 4 studies. Two of these reported resistance to quinolones, but at a low rate (2%). Third-generation cephalosporin resistance was also assessed in 2 studies, and sulfonamide resistance was assessed in a single study; both were found to be below 10%. There was a lack of consistency between risk factors assessed for their impact on AR E. coli with only the effects of age, sex and antibiotic use being reported in more than 1 study. The 2 studies that assessed the impact of antibiotic use found no increased risk of AR E. coli carriage despite both reporting on recent (within 4 months) use. The single study that assessed carriage of AR E. coli among household members found that infants carrying AR E. coli were more likely to have a mother who also carried AR E. coli (53%) compared with AR E. coli negative infants (6%). One study assessed the effects of multiple factors associated with day-care centre hygiene and found that children attending day-care centres with increased hand-washing practices and with enforced policies regarding outbreaks (ie, not allowing attendance of ill children and reporting illness to public authorities) had a decreased risk of carrying AR E. coli. One study investigated foreign travel by the child and household members and found that foreign travel by the child (with household members) was associated with a 185%–594% increase in AR E. coli carriage.
**Overall conclusion**	Antibiotic use is a major promoter of antibiotic resistance in individuals. However, less than half of the studies included in this review that reported on the risk associated with antibiotic use found a significant effect on carriage of AR bacteria in children in the community. Despite worrying increases in extended-spectrum cephalosporin-resistant Gram-negative bacteria, only 4 studies assessed risk factors associate with carriage of AR E. coli in healthy children in the community. This is even more surprising considering the ease of sample collection and the exponential growth in research into the microbiome, including the infant and child gut microbiome, over the past decade
**Author, year**	**O'Brien 2019 [51]**
**Type of review**	Systematic review
**AMR outcome category**	Humans
**Objectives/ Aim of study**	To summarise the existing evidence on the prevalence of antimicrobial resistance following mass azithromycin distribution for trachoma and define future research priorities
**AMR definition**	Carriage

**Population specifics**	Treatment population in one study was non-pregnant residents of 6 months of age or older and in another study all residents. The sample population of the first study were children aged < 3 years, children reporting diarrheal symptoms and randomly selected children aged 1 month to 5 years and of the second study randomly selected parent-child pair with the children aged < 5 years.
**Setting specifics**	Communities in Tanzania
**Overall results**	Macrolide resistance after azithromycin distribution was reported in three (E. coli, S. Pneumoniae and S. Aureus) of the five organisms studied (E. coli, S. Pneumoniae, S. Aureus, C. Trachomatis and P. Falciparum).
**E. coli specific results**	We included three studies on E coli, based in Tanzania: two examined the same population of four treated communities included in a cohort study and the third used a cross-sectional design in 32 communities. The two studies from the same population assessed resistance at baseline and at 1, 3, and 6 months after a single distribution of azithromycin and included an untreated control group. The other study assessed resistance 48 months after four annual distributions of azithromycin and included neither a baseline assessment nor an untreated control group. The two studies from the same population each included 160 children younger than 3 years, with one of the studies also including an additional sample of children reporting diarrhoea. The other study included 1048 randomly selected children aged 1–5 years. Resistance was assessed with E-test in two studies and Kirby-Bauer disk diffusion in the third. All studies on E coli found macrolide resistance after treatment. One of the cohort studies reported a short-term increase in macrolide resistant E coli isolates immediately after the single treatment, followed by decreasing resistance over 6 months, with the final assessment at 6 months showing macrolide resistance greater than baseline levels. At all post-treatment time points, treated communities in this study had an increased odds of carriage of macrolide-resistant isolates compared with untreated communities (1 month aOR 11·2, 95% CI 7·1–17·6, p<0·001; 3 month aOR 10·6, 3·8–29·9, p<0·001; 6 month aOR 4·8, 1·5–14·9, p<0·001).
**Overall conclusion**	Although there has been enormous success in controlling ocular C. trachomatis infection with mass azithromycin distribution, available evidence suggests that these distributions select for macrolide resistance in some potentially pathogenic organisms
**Author, year**	**Ramblière 2021 [52]**
**Type of review**	Systematic review
**AMR outcome category**	Humans
**Aim of study**	(i) To provide a descriptive overview of MDA/SDA interventions implemented in LMICs, including indications, targeted populations, antibiotics used and modes of administration; and (ii) to investigate their potential impact on AR.
**AMR definition**	Colonization
**Population specifics**	HIV-infected adults and HIV-exposed children (0-15 months old)
**Setting specifics**	Countries defined as LMIC

**Overall results**	Only 14 (39%) out of 36 studies evaluated antibiotic resistance after mass and systemic administration of amoxicillin, azithromycin, ampicillin and co-trimoxazole. Despite co-trimoxazole resistance being high at baseline for E. coli (>50%) and S. Pneumoniae (>75%), this increased further in several populations receiving co-trimoxazole MDA/SDA.
**E. coli specific results**	Phenotypic AR of faecal E. coli in HIV-exposed or -infected adults were similar at 24 weeks for both groups. Compared to placebo, higher proportions of E. coli resistance to ampicillin (OR=10·2; P<0·001), chloramphenicol (OR=7·8; P<0·001), ciprofloxacin (OR=17·1; p=0·006) and nalidixic acid (OR=26·4; P=0·001) were found. In HIV-exposed but uninfected infants, proportion of E. coli resistant to co-trimoxazole was higher in recipients of co-trimoxazole compared with placebo at 3 (94% vs. 51% (P<0·0001)) and 6 months (84% vs. 57% (P=0·01)). HIV patients with CD4 cell counts lower than 350 cell/mm3 were at higher risk of E. coli resistant co-trimoxazole 54% (vs 29% in control) and reached 100% (vs 53%) at 12 months. Resistance were also higher compared to baseline with ampicillin (from 74% to 100%), amoxicillin/clavulanic acid (from 33% to 100%) and ceftriaxone (from 2% to 54%) in this group.
**Overall conclusion**	However limited, findings are consistent with the expectation that MDA/SDA interventions lead to greater AR prevalence, especially following cotrimoxazole and azithromycin administration.
**Author, year**	**Truong 2022 [53]**
**Type of review**	Systematic review
**AMR outcome category**	Humans
**Aim of study**	Investigate the impacts of oral tetracycline class antibiotics on the development of AMR in normal human flora. A secondary objective was to investigate impact on resistance to non-tetracycline class antibiotics.
**AMR definition**	Acquisition
**Population specifics**	Adults using tetracycline daily and oral
**Setting specifics**	Human flora
**Overall results**	All seven articles evaluated the impact of oral tetracyclines on outcomes relating to the burden of tetracycline-resistant isolates. All articles also suggested evidence of varying levels of tetracycline resistance at baseline for both the intervention and comparator arm. In general, five studies suggested that oral tetracycline use was associated with an increased burden of tetracycline-resistant isolates in the assessed normal flora. Specifically, all three studies that investigated subgingival flora (n=20 each) demonstrated a relatively small increase in the percentage of isolates resistant to tetracyclines during 2 weeks of antibiotic therapy.<br>Only one article evaluated the impact of oral tetracyclines on tetracycline MICs. Brillet al. illustrated that the MIC of the upper respiratory flora significantly increased by a factor of 3·74 in individuals who took 100 mg of doxycycline daily for 13 weeks, compared with those who took placebo. It was also demonstrated that those who took this doxycycline regimen were 5·77 times more likely (95% CI: 1·40–23·74, p=0·02) to have doxycycline-resistant isolates than individuals who took the placebo.

	Three of the seven included studies evaluated the impact of oral tetracyclines on outcomes relating to non-tetracycline antibiotics. Overall, these studies demonstrated that oral tetracyclines had negligible effects on non-tetracycline resistance in commensal E. coli in the gastrointestinal tract. However, one 1978 study among Peace Corps volunteers showed a transient increase in multiply resistant commensal and pathogenic E. coli in stool isolates after 3 weeks of doxycycline compared with no intervention, with a return to baseline 2 weeks after treatment
**E. coli specific results**	Two studies demonstrated an increase in tetracycline-resistant commensal Escherichia coli in the gastrointestinal tract, based on cultures of stool specimens. However, Sacket al. reported that the number of both commensal and pathogenic *E. coli* isolates resistant to tetracycline returned to baseline 2 weeks after taking 100 mg (200 mg on Day 1) of doxycycline daily for 3 weeks.
**Overall conclusion**	Although the effects are modest and transient, limited data from small prospective studies found that oral tetracyclines for 2 - 18 weeks increased resistance to tetracycline class antibiotics in the subgingival, gastrointestinal and upper respiratory flora, but not in skin flora, when compared with non-tetracycline exposed controls. Conversely, oral tetracyclines had no significant effect on resistance to other non-tetracycline class antibiotics in commensal E. coli and propioni bacteria (now renamed cuti bacteria)
**Author, year**	**Voor in 't Holt 2020 [54]**
**Type of review**	Meta-analysis
**AMR outcome category**	Humans
**Aim of study**	Identify the carriage rates of multidrug-resistant Enterobacterales (MDR-E) among returning travellers, to identify microbiological methods used, and to identify the leading risk factors for acquiring MDR-E during international travel.
**AMR definition**	Acquisition, carriage
**Population specifics**	Foreign travellers with faecal Enterobacterales carriage without signs of infections when performing the screening: travellers visiting a travel or vaccination clinic, hospital staff and contacts, healthcare students, business travellers and Hajj pilgrims.
**Setting specifics**	Worldwide travel from people living in the community in Western Europe, North America, Japan and Australia
**Overall results**	See E. coli specific results
**E. coli specific results**	We identified 12 studies describing protective and/or risk factors for acquiring MDR-E during travel, obtained from multivariable analyses. The highest OR was reported for the risk factor Travel Diarrhoea (TD) (OR =31·00, 95% CI = 2·70 to 358·10). Examples of identified protective factors were handwashing with soap before meals, a beach holiday, tap water consumption, and travel to various countries compared to traveling to Asia. All identified risk factors, protective factors, and factors identified as non-significant in multivariable models were grouped into 8 categories. For these 8 categories, 7 meta-analyses were performed. For the category length of stay, three factors were identified. However, for one factor the confidence interval was missing. Therefore, for the category length of stay no meta-analysis was performed. The factor antibiotic use during travel was most frequently described (n= 13 times identified).The risk factor with highest pooled OR was found to be travel to Southern Asia (pooled OR = 14·16, 95% CI =5·50 to 36·45), followed by antibiotic use during travel (pooled OR = 2·78, 95% CI = 1·76 to 4·39) and TD (pooled OR = 2·02, 95% CI = 1·45 to 2·81). The factors behaviour

	during travel, food consumption during travel, male gender, and older age were identified as non-significant factors. Publication bias indicators Begg-Mazumdar (Kendall's tau) and Egger both showed a statistically significant result in the following meta-analyses: travel to Southern Asia, and older age.
**Overall conclusion**	Risk of acquiring MDR-E while travelling increases depending on travel destination and if antibiotics are used during travel.
**Author, year**	**Willems 2020 [55]**
**Type of review**	Meta-analysis
**AMR outcome category**	Humans
**Aim of study**	Examine the association of use of acid suppressants with the risk of colonization with MDROs and to perform a meta-analysis of current evidence
**AMR definition**	Colonization, carriage, infection
**Population specifics**	Adult acid suppressant users
**Setting specifics**	travel clinics, community
**Overall results**	See E. coli specific results
**E. coli specific results**	Meta-analysis of the 4 community-based studies on colonization with MDROs after acid suppressant use showed an association (OR = 1·41; 95% CI, 1·07-1·87; I2= 21%). However, meta-analysis of 4 travel-based studies yielded an OR with a very broad CI (OR = 1·11; 95% CI, 0·82-1·50; I2= 0%).
**Overall conclusion**	This systematic review and meta-analysis showed that the use of acid suppressants (mainly PPIs or H2RAs) is associated with a 75% increase in the odds of intestinal MDRO colonization, both in the community and in the health care setting.
